# Antagonistic control of the turnover pathway for the global regulatory sRNA CsrB by the CsrA and CsrD proteins

**DOI:** 10.1093/nar/gkw484

**Published:** 2016-05-27

**Authors:** Christopher A. Vakulskas, Yuanyuan Leng, Hazuki Abe, Takumi Amaki, Akihiro Okayama, Paul Babitzke, Kazushi Suzuki, Tony Romeo

**Affiliations:** 1 Department of Microbiology and Cell Science, University of Florida, Gainesville, FL 32611-0700, USA; 2 Graduate School of Science and Technology, Niigata University, Niigata 950-2181, Japan; 3 Department of Biochemistry and Molecular Biology, Center for RNA Molecular Biology, Pennsylvania State University, University Park, PA 16802, USA; 4 Department of Applied Biological Chemistry, Faculty of Agriculture, Niigata University, Niigata 950-2181, Japan

## Abstract

The widely conserved protein CsrA (carbon storage regulator A) globally regulates bacterial gene expression at the post-transcriptional level. In many species, CsrA activity is governed by untranslated sRNAs, CsrB and CsrC in *Escherichia coli*, which bind to multiple CsrA dimers, sequestering them from lower affinity mRNA targets. Both the synthesis and turnover of CsrB/C are regulated. Their turnover requires the housekeeping endonuclease RNase E and is activated by the presence of a preferred carbon source via the binding of EIIA^Glc^ of the glucose transport system to the GGDEF-EAL domain protein CsrD. We demonstrate that the CsrB 3′ segment contains the features necessary for CsrD-mediated decay. RNase E cleavage in an unstructured segment located immediately upstream from the intrinsic terminator is necessary for subsequent degradation to occur. CsrA stabilizes CsrB against RNase E cleavage by binding to two canonical sites adjacent to the necessary cleavage site, while CsrD acts by overcoming CsrA-mediated protection. Our genetic, biochemical and structural studies establish a molecular framework for sRNA turnover by the CsrD-RNase E pathway. We propose that CsrD evolution was driven by the selective advantage of decoupling Csr sRNA decay from CsrA binding, connecting it instead to the availability of a preferred carbon source.

## INTRODUCTION

Small non-coding RNAs (sRNAs) have emerged as important regulators of gene expression throughout the biological world, including the prokaryotes (1–4). While the majority of bacterial sRNAs appear to act by base-pairing to mRNA targets, a few protein binding sRNAs, which mimic the nucleic acid substrates of proteins, have broad impacts on regulation and physiology (5–8). Prominent among these are the sRNAs that govern the carbon storage regulatory (Csr) or repressor of stationary phase metabolites (Rsm) system of bacteria, which oversees major shifts in the bacterial lifestyle and the expression of virulence factors of pathogens (5,6,9,10). The CsrA/RsmA/RsmE proteins of this system act by binding to mRNA and altering its structure or the binding of ribosomes or proteins that occupy overlapping or nearby binding sites, thereby affecting translation, RNA stability and/or transcription termination (6,11–15). CsrA binding sites reside in the untranslated leader of target mRNAs, as well as non-coding sRNAs, such as CsrB of *Escherichia coli*, which sequester CsrA away from mRNA (16–19). CsrA binding sites consist of a GGA motif flanked by conserved sequences, typically CAGGA(U/A/C)G in CsrB (17). CsrB from *E. coli* contains 22 GGA sequences, most of which are inferred to serve as CsrA binding sites (16,18). High-affinity CsrA binding sites are found within the single-stranded loops of RNA hairpins, although binding can occur in the absence of secondary structure (17). The CsrA protein is a homodimer containing two identical RNA binding surfaces, which allow it to bridge two binding sites in target RNAs (20,21). Accordingly, most of its known RNA targets contain two or more CsrA binding sites, although exceptions exist (22).

CsrA levels increase modestly as *E. coli* cells undergo the transition from the exponential to stationary phase of growth (23) and *csrA* gene expression is subject to complex autoregulation (24). Nevertheless, it appears that alterations in the levels of CsrA inhibitory sRNAs are largely responsible for governing CsrA activity in response to environmental conditions. Evidence suggests that CsrA-inhibitory sRNAs probably occur throughout the *γ-Proteobacteria*, although studies of their regulation are limited to a few species (25,26).

**Figure 1.**
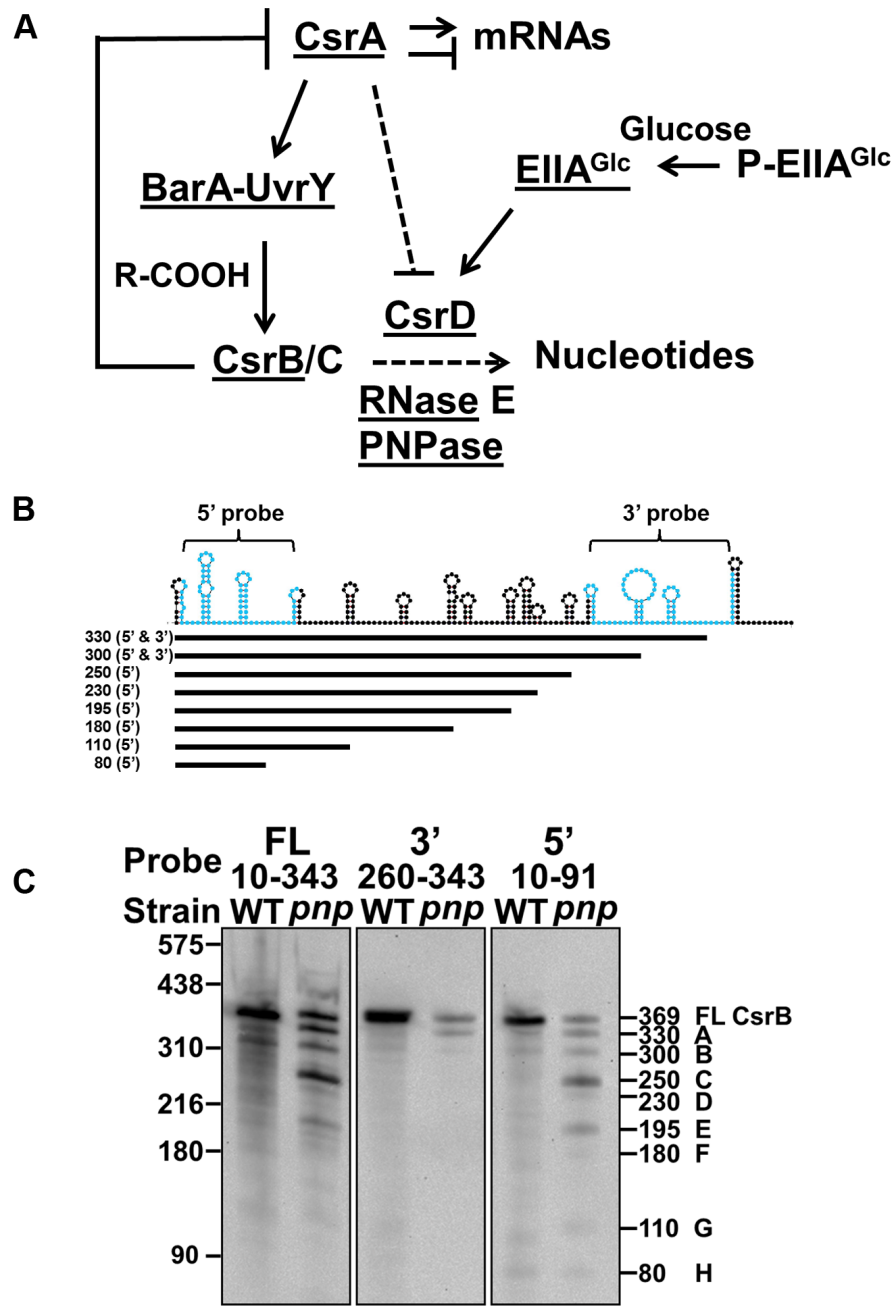
CsrB decay products in MG1693 (WT) and isogenic *pnp* mutant *Escherichia coli*. (**A**) Csr regulatory circuitry relevant to the present study (18,27,28,33,34). (**B**) The predicted secondary structure of CsrB generated by mfold (76). The regions of CsrB complementary to the 5′ and 3′ riboprobes are indicated in blue (top). CsrB decay intermediates were drawn in comparison to the full length RNA, with the length and antisense riboprobe that detected each RNA indicated to the left (bottom). (**C**) Northern blots using antisense RNA probes complementary to the full length (FL) (nt 10–343), 3′ end (nt 260–343) and 5′ end (nt 10–91) sequences of CsrB. CsrB decay intermediates were detected by comparing total RNA isolated from wild-type MG1693 (WT) and *pnpΔ683* (*pnp*) mutant strains. The sizes in nt of RNA standards (left) and the approximate lengths of RNA decay intermediates are indicated (right). The RNA species and nomenclature (A to H) for each cleavage product are indicated (right).

Csr sRNA levels are regulated in part by a conserved bacterial two-component signal transduction system (TCS) (Figure 1A), referred to as BarA-UvrY in *E. coli* (6,26,27). BarA is a membrane-bound sensor kinase that responds to carboxylate-containing metabolites such as acetate and formate (28). This causes BarA to phosphorylate the response regulator, UvrY, which activates transcription from the *csrB* and *csrC* promoters (26,29). Other influences on CsrB/C synthesis include CsrA itself (23,26,30), the stringent response components ppGpp and DksA (26,31), and two DeaD-box RNA helicases, DeaD and SrmB, which act by distinct mechanisms (32).

Csr sRNA levels in *E. coli* are also regulated by changes in their stability. We recently reported that CsrB/C decay is activated by the presence of a preferred carbon source, e.g. glucose (33). This physiological response is accomplished via the binding of the unphosphorylated form of EIIA^Glc^ protein of the PEP:carbohydrate phosphotransferase system (PTS), which predominates when glucose is being actively transported by this system, to the EAL domain of CsrD. This interaction apparently leads to allosteric activation of CsrD. The CsrB/C RNAs have short half-lives (∼2–4 min) in exponentially growing wild-type (WT) strains, but are stabilized in strains lacking CsrD or EIIA^Glc^ (33,34). Through its effect on CsrB/C turnover, CsrD regulates the expression of genes and physiological processes that are controlled by CsrA (34). CsrD is not a nuclease, and CsrB/C turnover requires the major endonuclease RNase E and the exonuclease PNPase, which eliminates RNase E cleavage products (34). CsrD is a predicted inner membrane protein containing GGDEF and EAL domains, which typically catalyze the synthesis and turnover, respectively, of the secondary messenger c-di-GMP (34,35). However, CsrD lacks critical catalytic amino acid residues of these domains and does not appear to synthesize, hydrolyze or mediate a response to c-di-GMP (34). While available evidence suggests that CsrD might facilitate RNase E cleavage by interacting with CsrB/C RNAs, the binding of CsrD to these RNAs *in vitro*, even in the presence of EIIA^Glc^, appears to be non-specific (33,34).

CsrB/C in both *E. coli* and *Salmonella enterica* Typhimurium are unstable during rapid growth (23,34,36), and while CsrA modestly represses *csrD* expression, it has little or no effect on CsrB/C decay rates (23,34). In stark contrast, the Csr (Rsm) sRNAs of *Pseudomonas sp*. are much more stable, with half-lives reported from ∼20 min to >60 min (37,38). Furthermore, elimination of the CsrA homolog in *Pseudomonas sp*. greatly reduces the stability of these sRNAs, possibly because CsrA binding to these RNAs protects them from ribonuclease cleavage (38,39). The distinct sRNA decay responses to CsrA-family proteins in these species have not been explained. However, unlike *E. coli* and its relatives, the pseudomonads possess no apparent CsrD ortholog.

Here, we have begun to investigate the molecular basis of CsrB decay. We found that RNase E cleavage just upstream of the intrinsic terminator of CsrB is required before decay can proceed in the 5′ direction. A deletion corresponding to this necessary cleavage site (NCS) virtually eliminated turnover. In addition, CsrA binding to two GGA sites located immediately upstream from the NCS provided protection against RNase E attack both in a *csrD* mutant and *in vitro*. However, CsrA did not protect CsrB from turnover in the *csrD* WT strain. The structure of CsrA-bound and free CsrB RNA in the RNase E-susceptible region was investigated by in line probing studies. Our findings support a model wherein sRNA turnover is antagonistically determined by two non-nucleolytic proteins: CsrD facilitates CsrB turnover by counteracting the protection afforded by CsrA binding near the 3′ end of this RNA. We propose that CsrD evolved in a subset of the *γ-Proteobacteria* to become a device for decoupling Csr sRNA turnover from the direct influence of CsrA binding and governing it instead according to the availability of a preferred source of carbon nutrition.

## MATERIALS AND METHODS

### Bacterial strains and culture conditions

Bacterial strains used in this study are described in Supplementary Table S1. *E. coli* strains were maintained on LB medium containing the following antibiotics as necessary: ampicillin (100 μg ml^−1^), tetracycline (15 μg ml^−1^), kanamycin (50 μg ml^−1^), gentamicin (10 μg ml^−1^), streptomycin (10 μg ml^−1^) and chloramphenicol (25 μg ml^−1^). Bacteria were routinely grown using the following protocol unless otherwise indicated: LB medium (2 ml) was inoculated with bacterial strains from frozen glycerol stocks and cultures were grown with shaking at 37°C overnight. Thymine (50 μg ml^−1^) was added to LB medium for growth of strains containing the *thyA715* allele (MG1693 and its derivatives). Stationary phase cultures were used to inoculate LB medium and growth was monitored (OD_600_). Cultures were grown with shaking (250 rpm) at 37°C, and samples were taken for RNA extraction at late exponential phase (OD_600_ 0.8) unless otherwise indicated. For artificial induction of CsrB expression, cultures were grown with shaking (250 rpm) at 37°C to mid-exponential phase (OD_600_ 0.6), at which time arabinose (final concentration 0.25%) was added. Cultures were incubated an additional 20 min at 37°C with shaking (250 rpm) and RNA was subsequently extracted. Kornberg medium (1.1% K_2_HPO_4_, 0.85% KH_2_PO_4_, 0.6% yeast extract and 0.5% glucose), which permits a high rate of CsrB turnover (34) was alternatively used to grow bacterial cultures, as indicated.

### Plasmid construction

Primers used for plasmid construction, their sequences and relevant restriction sites are identified in Supplementary Tables S2 and 3. The CsrB expression plasmid p1VR147f (and its derivatives) was created by polymerase chain reaction (PCR) amplification of the *araC-*P*_araB_* cassette (−1250 bp to +1 relative to the start of P*_araB_* transcription), and the CsrB sequence, and cloning the resulting fragments into pAH125. Integration of the CRIM plasmid pAH125 derivatives into the chromosome at the λ*att* site was accomplished using previously published procedures (40). Mutations in the p1VR147f plasmid were introduced by site-directed mutagenesis using the Quickchange II XL kit (Stratagene). The CsrA expression vector p2VR112 was created by PCR amplification of the *csrA* gene (300 bp upstream of the start codon to the stop codon) and cloning the resulting fragment into pBR322. The carboxy-terminal RNase E_HIS_ expression vector p1VR22 was created by PCR amplification of the *rne* gene and cloning the resulting fragment into pET-24a (Novagen).

### Creation of *csrA::gm*

The kanamycin resistance marker in the *csrA::kan* disrupted strain (41) was replaced with a gentamicin resistance marker using the λ Red recombinase gene replacement method (42) to maintain compatibility with the CsrB expression cassettes used in this study. The *csrA::gm* DNA fragment used for gene replacement was created by amplification of the gentamicin gene from the *E. coli* / *Pseudomonas aeruginosa* shuttle vector pJN105 (43) using a 5′ primer that includes the constitutive P_tac_ promoter (44) and a 3′ primer that contains 40 nt of sequence that anneal immediately downstream of the kanamycin marker in the *csrA::kan* allele. The resulting PCR product was diluted and used as template in a second reaction using the same 3′ primer and a different 5′ primer that anneals just downstream of the truncated *csrA* gene in the *csrA::kan* disrupted allele. The resulting PCR product was purified and used to replace the *csrA::kan* allele with the λ red method as described previously (42). Gentamicin-resistant colonies were picked and the correct mutants were verified with PCR and sequencing.

### Purification of CsrA

*Escherichia coli* Tuner (DE3) cells carrying pCSB12 (17) were grown at 30°C in LB containing ampicillin to an OD_600_ of 0.7, at which time 1 mM isopropyl-β-D-thiogalactopyranoside (IPTG) was added and the culture was incubated for an additional 2 h at 30°C. Bacteria were harvested by centrifugation and suspended in 20 mM Tris (pH 8.0), 300 mM NaCl and 20 mM imidazole supplemented with a protease inhibitor cocktail (Roche). Cells were disrupted using a French pressure cell, and the CsrA_HIS_-containing lysates were cleared by centrifugation (20 000 *g*, 15 min, 4°C), and then subjected to Ni^2+^ affinity chromatography as previously described (17). Peak protein fractions were pooled and dialyzed against CsrA storage buffer (20 mM Tris (pH 8.0), 150 mM NaCl, 1 mM dithiothreitol (DTT) and 50% glycerol). Aliquots of purified CsrA_His_ were flash frozen in liquid nitrogen and stored at −80°C. Protein concentrations were determined using the bicinchoninic acid assay (Pierce Biotechnology) with bovine serum albumin as the protein standard.

### Purification of RNase E

*Escherichia coli* Tuner (DE3) cells carrying p1VR22 were grown at 30°C in LB containing kanamycin to an OD_600_ of 0.5, at which time 1 mM IPTG was added and the culture was incubated for an additional 2 h at 30°C. Bacteria were harvested by centrifugation and suspended in 20 mM Tris (pH 7.9), 150 mM NaCl, 2 mM DTT, 8 M urea and 20 mM imidazole supplemented with a protease inhibitor cocktail (Roche). Cells were disrupted by sonication, and the RNase E_HIS_-containing lysates were cleared by centrifugation (20 000 *g*, 15 min, 4°C), subjected to denaturing Ni^2+^ affinity chromatography as previously described (45). Peak protein fractions were pooled and dialyzed overnight at 4°C against RNase E storage buffer [20 mM Tris (pH 7.9), 250 mM KCl, 10 mM MgCl_2_, 2 mM DTT and 50% glycerol]. Aliquots of purified RNase E_HIS_ were stored at −20°C. Protein concentrations were determined using the bicinchoninic acid assay (Pierce Biotechnology) with bovine serum albumin as the protein standard.

### RNA decay analysis

*Escherichia coli* strains were grown in LB at 37°C to mid-exponential phase (OD_600_-0.6) and rifampicin was added to a final concentration of 200 μg ml^−1^. At various time points after rifampicin addition, 0.67 ml of the bacterial culture was added to two volumes of RNAprotect Bacteria Reagent (Qiagen) and incubated for 5 min at room temperature. Total cellular RNA was subsequently purified with the RNeasy Mini Kit (Qiagen) and CsrB RNA was detected by northern blotting. Densitometry was performed using Quantity One image analysis software (Bio-Rad), and semi-log decay curves were generated using Prism (GraphPad).

### Northern blotting

*Escherichia coli* cultures were grown as indicated and total cellular RNA was prepared and isolated using phenol:ethanol (10:90 by vol.) and the RNeasy mini kit (Qiagen) or using RNAprotect Bacterial Reagent (Qiagen) along with MasterPure RNA Purification Kit (Epicentre). For agarose gel electrophoresis, the RNA was mixed with two volumes of denaturing loading buffer (20 mM MOPS pH 7.0, 5 mM sodium acetate, 2 mM ethylenediaminetetraacetic acid (EDTA), 50% formamide, 6% formaldehyde and 10% glycerol) and samples were denatured at 65°C for 10 min and then immediately placed on ice. Denatured RNA samples (1 μg) were loaded onto 1.5% agarose MOPS (20 mM MOPS pH 7.0, 5 mM sodium acetate and 2 mM EDTA) gels containing 2% formaldehyde. Separation of RNA on 6% polyacrylamide gels containing 7M urea was conducted as previously described (34). RNA was transferred to positively charged nylon membranes (Roche Diagnostics) by vacuum-assisted capillary transfer using the 785 Vacuum Blotter (Bio-Rad) or using a Trans-Blot SD semidry transfer cell (Bio-Rad) according to the manufacturer's instructions. RNA was fixed to the membrane by UV cross-linking or baking at 120°C. Blots were hybridized overnight at 60°C with a DIG-labeled antisense CsrB RNA probe and developed using the DIG Northern Starter kit (Roche Diagnostics) according to the manufacturer's instructions. The antisense CsrB RNA probes were transcribed *in vitro* using the DIG Northern Starter kit (Roche Diagnostics) from a PCR product, according to the manufacturer's instructions. Blots were imaged using the ChemiDoc XRS+ system (Bio-Rad) and RNAs were quantified using Quantity One image analysis software (Bio-Rad). Where applicable, rRNAs (16S and 23S) served as loading controls, and were detected by methylene blue staining.

### Mapping of CsrB decay intermediates by 3′ RACE

The isolation and detection of CsrB decay intermediates from PNPase mutant strain *pnpΔ683* were performed as described previously (34). Precise mapping of CsrB decay intermediates was executed by 3′ RACE, using a previously established method (46) with modification. Briefly, strain *pnpΔ683* was grown at 37°C until the transition to stationary phase, and total RNA was isolated and DNase I treated using the MasterPure RNA Purification Kit (Epicentre). The resulting total RNA was dephosphorylated with Antarctic Phosphatase (NEB) and ligated to an RNA adapter 5′-Phos-UUCACU GUUCUUAGCGGCCGCAUGCUC-InvdT-3′ (Integrated DNA Technologies). Ligated RNAs were used as template in a reverse transcription reaction using the ThermoScript RT-PCR system (Invitrogen) and primers that anneal to the RNA adapter and the 5′ end of *csrB*. Amplified cDNAs were separated on 5% agarose gels, gel-extracted and cloned into pCR^®^2.1-TOPO^®^ (Life Technologies). Plasmid DNA was extracted from bacterial transformants and submitted for sequencing.

### *In vitro* transcription and end-labeling of RNAs

RNAs were synthesized *in vitro* using the Megashortscript T7 kit (Ambion) following instructions from the manufacturer. RNA loading buffer (95% formamide, 18 mM EDTA and 0.025% sodium dodecyl sulphate (SDS), Xylene Cyanol and Bromophenol Blue) was added to the synthesis reactions followed by denaturation at 65°C for 10 min. Reactions were immediately placed on ice for 2 min and electrophoresed on 6–8% polyacrylamide gelelectrophoresis gels containing 7 M urea. Gels were stained with ethidium bromide, RNAs were visualized with UV irradiation and excised with a scalpel. RNAs were extracted from the gel by the addition of 0.5 ml extraction buffer (0.3 M sodium acetate, 1 mM EDTA and 0.2% SDS) and rocking at 25°C overnight. Extracted RNAs were isolated by phenol chloroform extraction followed by ethanol precipitation. Precipitated RNAs were dissolved in 10 μl of 10 mM Tris–HCl (pH 7.0) and the RNA concentration was determined by UV spectrophotometry. Purified RNAs (10 pMol) were 5′ end-labeled with ^32^P using the KinaseMax™ Kit (Ambion) and purified using the gel extraction and RNA purification procedure described above. RNA labeling efficiency was determined by liquid scintillation counting. Prior to *in vitro* assays, RNA samples were heated to 85°C for 3 min and cooled to 25°C over 20 min.

### In-line probing analysis

In-line probing experiments were performed as described previously (47), with some modification to facilitate the analysis of bound CsrA. Experiments were initiated by the addition of 0.01 nM ^32^P-labeled RNA (∼100 000 cpm) and CsrA to reactions containing 50 mM Tris–HCl (pH 8.3 at 20°C), 20 mM MgCl_2_ and 100 mM KCl. Reactions were incubated at 25°C for 43 h. Reaction volumes were adjusted to 0.3 ml with ddH_2_O and RNA was extracted using phenol-chloroform and ethanol precipitation. GlycoBlue™ (30 μg, Life Technologies) was used as a carrier for ethanol precipitation and the resulting dried precipitates were dissolved in in-line loading solution (5 M urea and 1.5 mM EDTA, pH 8.0). Samples were normalized by liquid scintillation counting and separated by electrophoresis on 8% acrylamide gels (19:1) containing 7 M urea. Gels were dried onto chromatography paper and subjected to autoradiography.

### *In vitro* RNase E cleavage assays

RNase E assays were performed by first incubating 0.05 nM RNA (≥25 000 cpm) at 90°C for 3 min in RNase E reaction buffer (25 mM Tris–HCl, pH 7.9, 5 mM MgCl_2_, 60 mM KCl, 100 mM NH_4_Cl, 15 mM DTT and 7.5% glycerol), followed by cooling to 25°C over 10 min. The reactions were then incubated at 25°C for 10 min in the presence or absence of CsrA. RNase E was added to 37.5 nM and reactions were incubated at 25°C for an additional 10 min. Reactions were stopped with two volumes of RNA loading buffer, incubated at 65°C for 10 min to inactivate CsrA_HIS_ and subsequently kept on ice prior to electrophoresis. Samples were separated by electrophoresis on 8% acrylamide gels (19:1) containing 7 M urea for 3 h at 45 Watts. Gels were dried onto chromatography paper and subjected to autoradiography.

### RNA structure prediction

RNA structure predictions were created with the mfold web server (http://mfold.rna.albany.edu/?q=mfold) using default folding parameters, except that the maximum distance allowed for paired bases was 30.

## RESULTS

### CsrB decay requires a site located immediately upstream of the transcription terminator

To better understand how CsrB decay is regulated, we first sought to determine the RNase E cleavage sites in this RNA. The full length CsrB RNA is stable in the absence of RNase E, while several decay intermediates accumulate in an *E. coli* strain that lacks PNPase (*pnpΔ683*) (34). The latter decay intermediates may have been directly generated by RNase E cleavage and/or may have undergone further degradation by other nucleases. Furthermore, the previous analyses did not determine which CsrB segments gave rise to the observed decay products. We therefore performed northern blotting experiments with RNA isolated from a PNPase mutant (*pnpΔ683*) using anti-sense riboprobes that hybridized to the 3′ end (260–343 nt), 5′ end (10–91 nt) or almost the complete sequence (10–343) of the RNA (Figure 1B). In total, we detected eight major CsrB decay intermediates, all of which could be visualized with the full-length and 5′ end CsrB riboprobes (Figure 1C). Only the longest decay intermediates were detectable using the 3′ end CsrB riboprobe (Figure 1C). These results collectively suggested that the major CsrB decay intermediates posses an intact 5′ end, and that decay may initiate near the 3′ end of CsrB. Primer extension analysis was used to confirm that the 5′ end of CsrB is identical in both the WT and *Δpnp683* mutant strains (Supplementary Figure S1).

**Figure 2.**
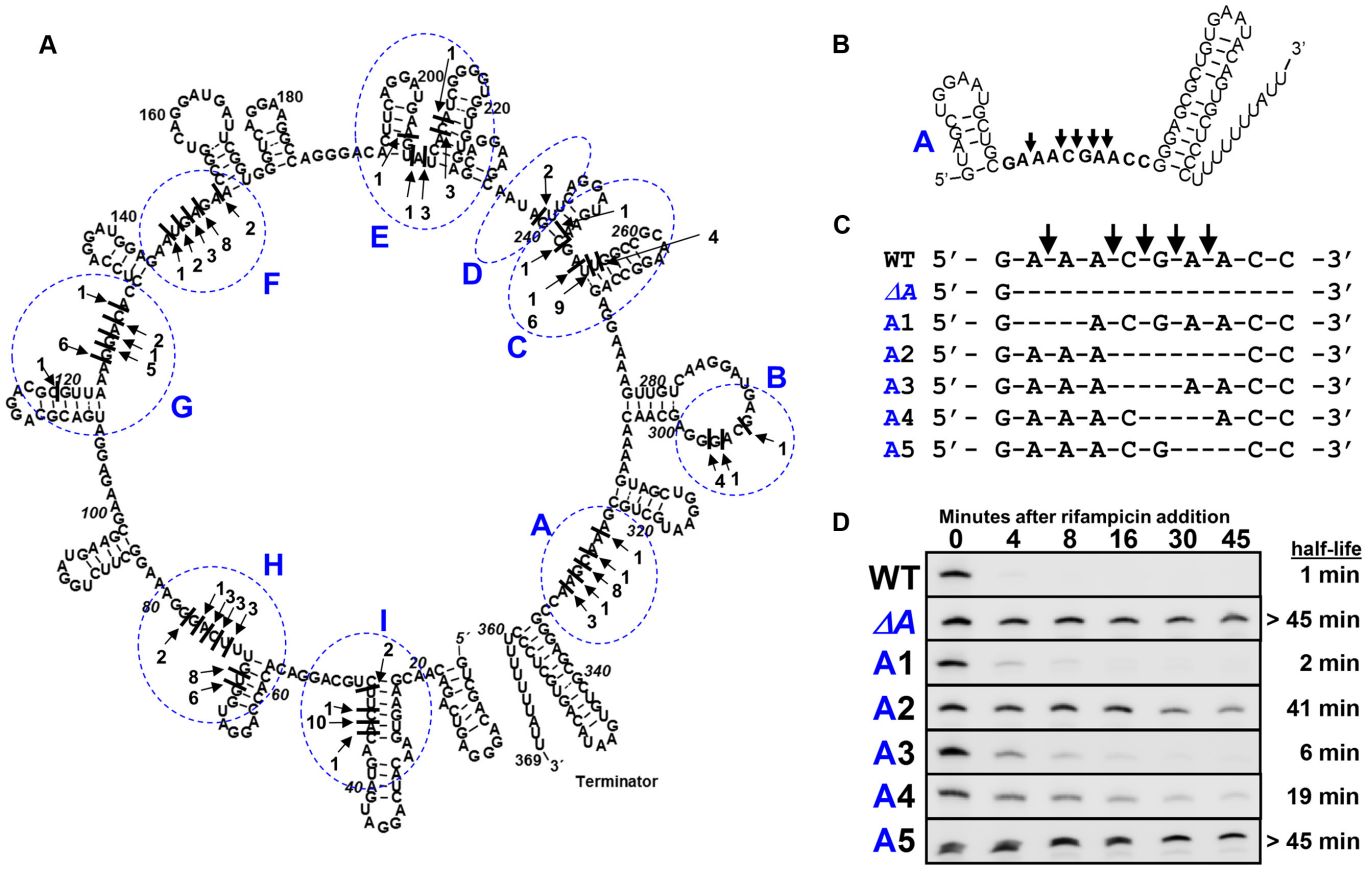
Mapping of CsrB decay intermediates with 3′ RACE. (**A**) The CsrB RNA structure as predicted with mFold. The 3′ ends of CsrB decay intermediates were mapped using 3′ RACE and indicated on the CsrB structure with black lines and marked with arrows. The frequency at which each 3′ end was isolated is indicated by the numbered arrows Mapped 3′ ends were found to be clustered in ∼10 nt segments, which are indicated with broken blue ovals and given letter designations (A-I) as shown. (**B**) The structure and sequence of cleavage region ‘A’ rotated and magnified. (**C**) Alignment of WT region ‘A’ (top) and various deletion mutations that were constructed in this region (below). (**D**) Northern blots comparing the stability of CsrB RNAs containing the WT and mutant region ‘A’ sequences, shown in panel C. Strain RGKSB837 harboring plasmids expressing WT or mutant CsrB were grown in Kornberg medium and treated with rifampicin at the transition to stationary phase growth. Calculated half-lives for these RNAs are indicated to the right. Based on these and other decay data, region ‘A’ is also referred to as the necessary cleavage site (NCS).

To more precisely determine CsrB cleavage sites *in vivo*, we mapped the 3′ ends of CsrB decay intermediates using 3′ rapid amplification of cDNA ends (3′ RACE) (Figure 2A). This analysis identified several distinct cleavage sites that were clustered in regions of sequence spanning 9 nt or less, which were designated A-I relative to the 3′-end of CsrB. As suggested by Northern blotting (Figure 1), the majority of CsrB decay intermediates contained an intact 5′ end and only the longest ones contained sequences from near the 3′ end. Therefore, we predicted that CsrB turnover initiates with cleavage in region ‘A’, located just upstream of the *csrB* transcription terminator (Figure 2A and B).

**Figure 3.**
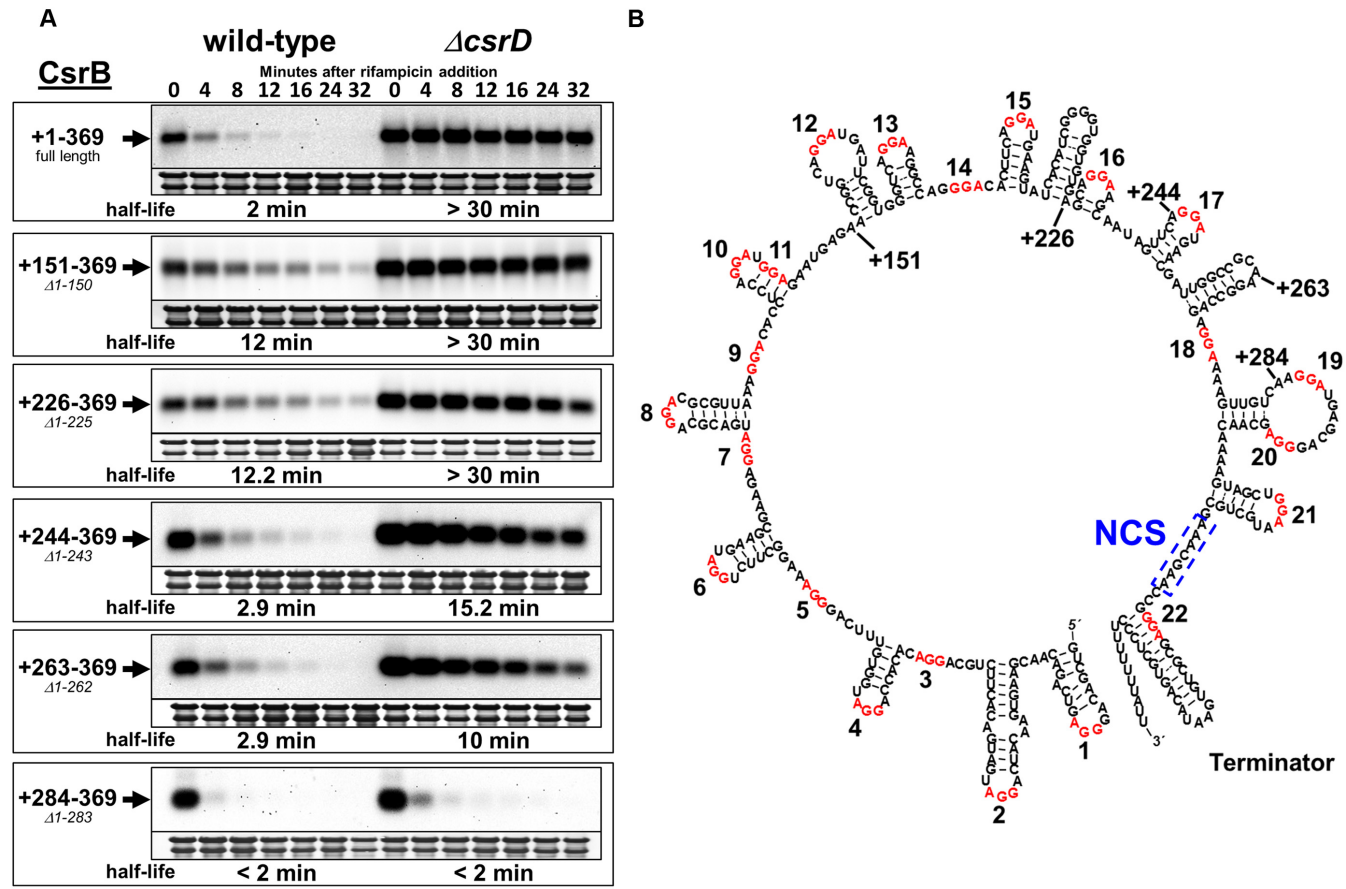
Decay properties of 5′ truncated CsrB RNAs. (**A**) *Escherichia coli* strains expressing the WT CsrB RNA (+1–369 ‘full length’) or 5′ truncated CsrB RNAs (+151–369 ‘*Δ1*–*150*’, +226*–*369 ‘*Δ*1–225’, +244*–*369 ‘*Δ*1–243’, +263–369 ‘*Δ*1–262’ and +284–369 ‘*Δ*1–283’) under *araP_BAD_* transcriptional control from the phage λ attachment site. Expression was induced by addition of arabinose at the mid-exponential phase of growth (OD_600_ of 0.6), and rifampicin RNA decay assays were performed 10 min following induction. Samples were collected for RNA isolation at the indicated time points after rifampicin addition. The 23S and 16S rRNAs were detected by methylene blue staining and represent RNA loading controls. Half-lives were calculated by non-linear regression analysis of best-fit decay curves. All strains were deleted for *csrB* at its native chromosomal locus and curves for the WT and isogenic *csrD* deletion strains are indicated. (**B**) Computer-generated structural prediction (mfold) for full-length CsrB RNA, with truncation sites indicated. GGA sequences, which may serve as CsrA binding sites, are shown in red and numbered sequentially in the 5′ to 3′ direction. The necessary cleavage site (NCS) (Figure 2) is boxed.

We furthermore reasoned that if CsrB turnover is initiated by cleavage(s) in region ‘A’, then deleting nucleotides in this region may stabilize full length CsrB (Figure 2B and C). Consistent with this hypothesis, a CsrB mutant RNA lacking the 9 nt of region ‘A’ (called *ΔA*) was very stable, with a half-life of >45 min (Figure 2C and D). CsrB mutants with other deletions in region ‘A’ were modestly (A1, A3) to strongly (A2, A5) stabilized relative to WT CsrB (Figures 2C and 3D). Most strikingly, a mutant lacking two adjacent adenine residues in the 3′-end of region ‘A’ was quite stable (*T*_1/2_ > 45 min), almost indistinguishable from the *ΔA* region mutant (Figure 2C and D). We also compared the pattern of CsrB decay products that accumulate over time from WT, *ΔA* and A5 mutant RNAs in a strain defective for PNPase (Supplementary Figure S2). These analyses showed only trace amounts of cleavage products from the two mutant RNAs, confirming the resistance of these RNAs to cleavage. Our previous findings demonstrated that RNase E activity is essential for CsrB decay, and in its absence, the full length CsrB RNA is stable (34). The observation that CsrB decay was virtually eliminated by a deletion of the cleavage region ‘A’ and greatly stabilized by elimination of two adjacent adenine residues within region ‘A’ indicates that the cleavages at other sites (‘B’ - ‘I’) are dependent on cleavage within region ‘A’. Because both RNase E (34) and region A (Figure 2D) are required for CsrB turnover, we infer that region A may constitute a necessary cleavage site (NCS) for RNase E to cause the turnover of CsrB *in vivo*.

### A CsrB segment that is subject to CsrD-dependent decay

Because CsrB decay requires CsrD in addition to RNase E (34), our next strategy was to identify RNAs containing the minimal sequences and/or structures necessary for CsrD-dependent decay before subjecting these RNAs to further studies. Because decay was inferred to initiate near the 3′ end of CsrB, we constructed a series of *csrB* alleles initiating at nucleotide +151, +226, +244, +263 or +284, and containing the remainder of the *csrB* gene through the terminator. The mutant *csrB* genes were expressed from the λ*att* locus under the control of the arabinose-inducible *araP*_BAD_ promoter. WT and *ΔcsrD* mutant *E. coli* strains containing *csrB* or its truncated derivatives were grown to mid-exponential growth phase, CsrB expression was induced with arabinose, rifampicin was added after 20 min and RNA decay was monitored. We found that a deletion of *csrD* resulted in the stabilization of all derivatives containing at least 107 nt of the CsrB 3′ segment, i.e. RNAs initiating at +151, +226, +244 and +263, indicating that the 5′ end of CsrB is not essential for the CsrD-dependent decay pathway (Figure 3). In contrast, an RNA comprised of only the 3′ terminal 85 nt (a deletion of nt 1–283) of CsrB was extremely unstable (half life < 2 min) in both *csrD* mutant and WT backgrounds. This result indicated that the 3′ 107-nt region of CsrB contains sequence and/or structural features that make it susceptible to CsrD-dependent degradation. In addition, something in the +263 to +283 segment affects susceptibility to CsrD-dependent turnover, because in its absence, turnover is rapid even in the absence of CsrD. Nevertheless, CsrB RNAs initiating at +151 or +226 nt were found to be stabilized in the *csrD* WT strain ∼6-fold relative to the full-length CsrB RNA, suggesting that features located within the 5′ end of CsrB can affect its degradation.

### CsrA affects the CsrB decay pathway *in vivo*

**Figure 4.**
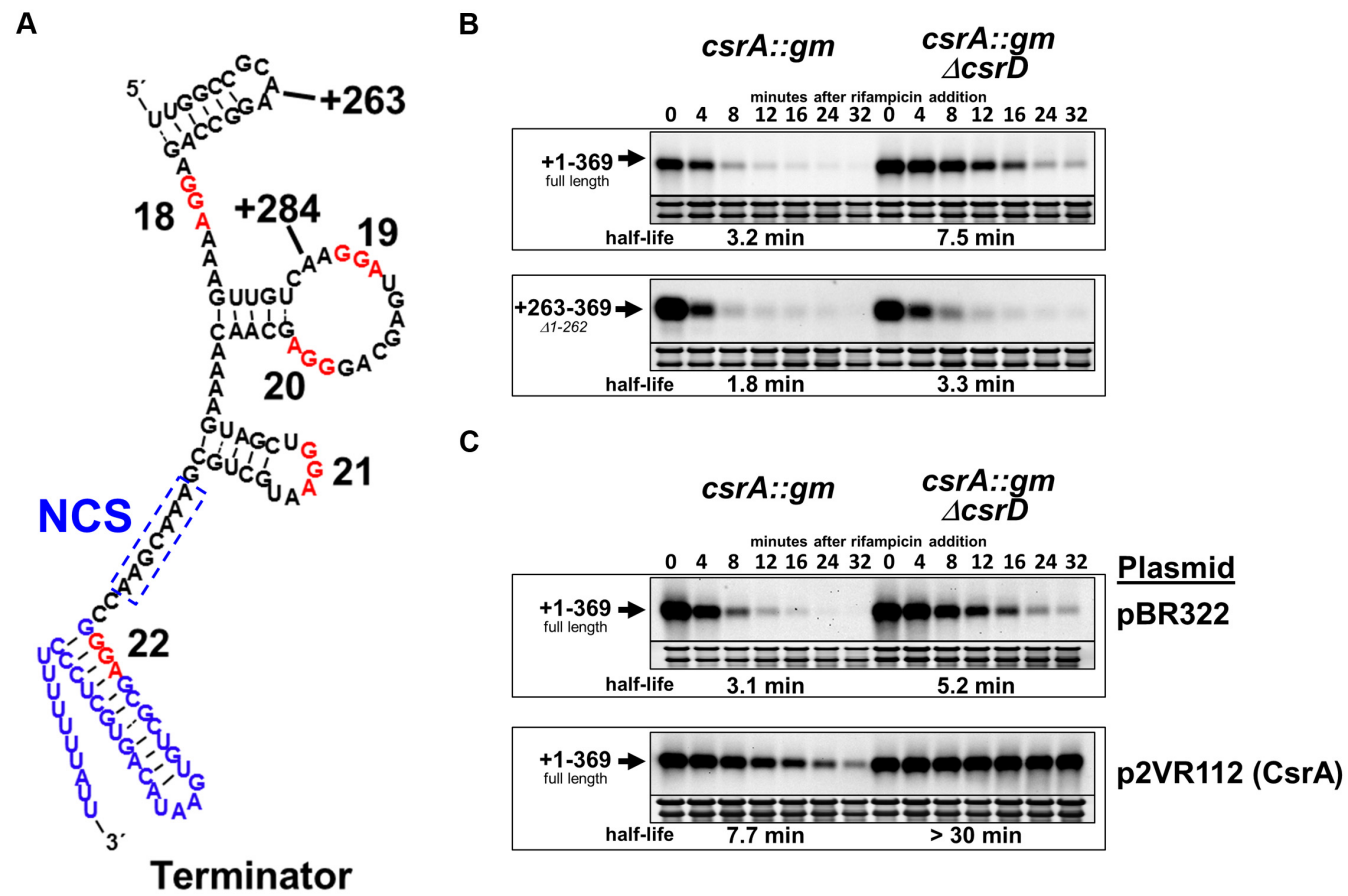
Mutation of *csrA* affects CsrD-dependent decay of CsrB. (**A**) Structure prediction of the 3′ region of CsrB that encompasses the +263 mutant RNA. The GGA sequences, which may represent CsrA binding sites and the transcription terminator are depicted with red and blue text, respectively. The necessary cleavage site (NCS) is boxed. (**B**) Northern blots comparing the stability of CsrB and the +263 truncated CsrB RNAs expressed in *csrA::gm* or *csrA::gm ΔcsrD* strains. (**C**) Northern blots analyzing CsrB stability with complementation of the *csrA::gm* mutation by the CsrA expression plasmid p2VR112 or a vector control (pBR322).

The truncation experiments described above showed that CsrD destabilizes a 3′ segment of CsrB that contains 107 nt of the original 369 nt sRNA (Figure 3). A computer-generated RNA structure prediction for this CsrB fragment revealed five GGA sequences, four of which are located in single stranded RNA and might represent CsrA binding sites, another within a strong transcription terminator hairpin and no other obvious sequence or structure elements (Figure 4A). Previous reports demonstrated that a *csrA* mutation had negligible to very modest effects on CsrB stability in *E. coli* (23,34). In contrast, in bacterial species that lack an apparent CsrD ortholog, CsrA-family proteins protect the Csr sRNAs from degradation (37–39). Therefore, we hypothesized that CsrA might protect CsrB RNA from degradation in *E. coli* in the absence of CsrD if CsrD functions specifically to facilitate the degradation of the sRNAs that may form or have formed a complex with CsrA. However, data from a previous study suggested that CsrB RNA is stable in a *csrA csrD* double mutant strain, in conflict with this hypothesis (34). Nevertheless, the previous experiments only analyzed CsrB stability for a relatively short period of time beyond the arrest of transcription with rifampicin (8 min) and appeared to show reduced CsrB levels at the final time point that was analyzed. For this reason we chose to reexamine the role of CsrA in the CsrB decay pathway.

The stabilities of full-length and the +263–369 truncated CsrB, whose turnover remains CsrD-dependent, were examined in *csrA::gm* and *csrA::gm ΔcsrD* double mutant backgrounds. The *csrA::gm* mutant allele is identical to that of the extensively studied *csrA::kan* disruption mutant (48), except that the kanamycin resistance marker that lies downstream of the disrupted *csrA* gene was replaced with the *aaC1* gentamicin resistance gene (49), which was genetically compatible with the various CsrB expression cassettes used in this study. As observed previously, the *csrA* mutation had only a minor effect on the stability of the full length CsrB RNA, which had a half-life of 2 min in the WT strain (Figure 3A) to 3.2 min in the *csrA::gm* mutant (Figure 4B). This was also observed for the +263 derivative, which had a half-life of 2.9 min in the WT (Figure 3A) to 1.8 min in the *csrA::gm* strain (Figure 4B). However, the introduction of *csrA::gm* into the *ΔcsrD* strain destabilized both the full-length CsrB, whose half-life decreased from >30 min (Figure 3A) to 7.5 min (Figure 4B) and the +263 CsrB, whose half-life changed from 10 min (Figure 3A) to 3 min (Figure 4B). The *csrA::gm* mutation did not completely eliminate the effect of CsrD on CsrB stability. CsrB was weakly stabilized (2-fold) by *csrD* deletion in a *csrA::gm* background (Figure 4B). A potential explanation for this result is that this *csrA* disruption is not a true null mutation, but encodes a mutant CsrA protein with greatly reduced RNA binding affinity (12). This *csrA* allele causes effects on gene expression that are similar to those of the null mutation, but unlike the null allele, it does not cause severely defective growth (50). The high affinity CsrA binding afforded by CsrB likely permits the mutant CsrA protein to bind weakly to CsrB and thus confer weak protection from decay (Figure 4B).

The half-life of CsrB was increased by the cloned *csrA* gene (p2VR112) in both the *ΔcsrD* and *csrD* WT strain backgrounds (Figure 4C). Furthermore, expression of CsrA in the *csrA::gm* strain moderately stabilized CsrB (∼ 3-fold), indicating that overproduction of CsrA can increase the stability of CsrB despite the presence of a functional CsrD protein.

### Mutation of two putative CsrA binding sites in CsrB relaxes the CsrD requirement for its turnover

**Figure 5.**
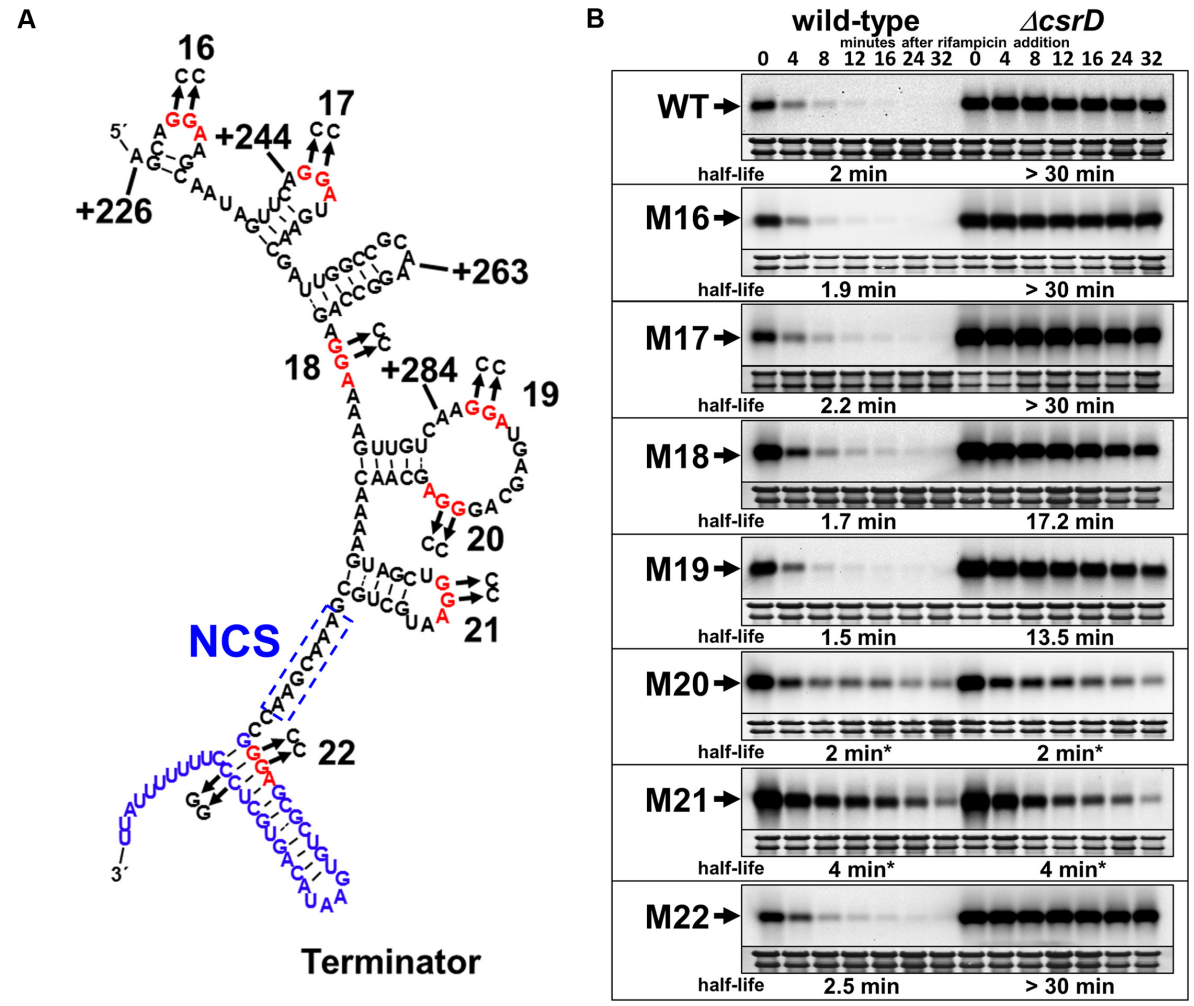
Mutation of CsrA binding sites near the 3′ end of CsrB eliminates CsrD-dependent stability control. (**A**) Structure prediction of the 3′ region of CsrB that encompasses the +226 mutant RNA. The GGA sequences representing potential CsrA binding sites and transcription terminator are indicated with red and blue text, respectively. The NCS is boxed. GGA to CCA mutations were introduced into the full length CsrB RNA and are numbered accordingly. (**B**) Northern blot decay analysis of full length CsrB (WT) and the GGA to CCA replacements that were introduced into the 3′ end of CsrB. CsrB RNAs M16 to M22 refer to mutant RNAs with GGA to CCA substitutions at sites 16–22. A CCA to GGA mutation was made to compensate for the site 22 GG to CC mutation in the transcription terminator to preserve the RNA hairpin structure.

If CsrA protects CsrB through a direct binding interaction, then mutation of CsrA binding sites within CsrB should destabilize the RNA. A sequence and structure analysis revealed seven GGA nucleotide sequences in the 3′ end of CsrB, five of which are present in the minimal +263 CsrB RNA (Figure 5A). Mutation of the conserved GGA nucleotide sequence within a CsrA binding site to CCA is sufficient to eliminate CsrA binding and regulation (51). Therefore, we used this strategy to eliminate CsrA binding to each of these seven potential sites *in vivo* (Figure 5A). These mutations were introduced into the gene encoding the full-length CsrB RNA, and RNA stability was analyzed in the presence and absence of CsrD. Because site 22 is located in the double stranded stem of the intrinsic terminator, we suspected that it does not represent an authentic CsrA binding site, which should only function in single stranded RNA (52). Nevertheless, we constructed the GGA to CCA replacement at site 22 along with a compensatory CC to GG mutation to preserve RNA structure and proper transcription termination. We observed that individual GG to CC mutations at sites 16 and 17, which are located upstream from the minimal +263 RNA, had no effect on CsrB stability in WT or *ΔcsrD* backgrounds (Figure 5B). This result is perhaps not surprising since these CsrA binding sites are found in a region of CsrB that is not essential for CsrD-dependent degradation (Figure 3). Mutations in sites 18 and 19 were weakly destabilizing in *ΔcsrD* background but had little to no effect in the *csrD* WT background (Figure 5B). In contrast, mutation of sites 20 and 21 strongly destabilized CsrB in *ΔcsrD*, both of which rendered the decay of CsrB RNA completely independent of CsrD. The latter mutations also appeared to alter the decay kinetics of the mutant RNAs relative to the WT CsrB, which in each case exhibited a slowly decaying component (Figure 5B). Because the later sites occur within the region of CsrB that was required for CsrD-dependent degradation (Figure 3) and relatively close to the NCS (Figure 2), it is perhaps not surprising that they affected turnover. Altogether, our findings suggested that CsrA protects CsrB from cleavage by RNase E at the NCS and that CsrD is required to overcome this CsrA-mediated protection.

### Analysis of CsrB structure and CsrA binding sites using in-line probing

CsrA-CsrB binding studies have been performed previously, but a limitation of these studies is that they did not analyze the specific CsrA binding sites within CsrB (16,18). Our *in vivo* experiments indicated that two predicted CsrA binding sites in the 3′ end of CsrB specifically contribute to the regulation of its turnover. We therefore wanted to know if CsrA actually binds to these predicted sites, and the consequences of CsrA binding for CsrB RNA structure.

**Figure 6.**
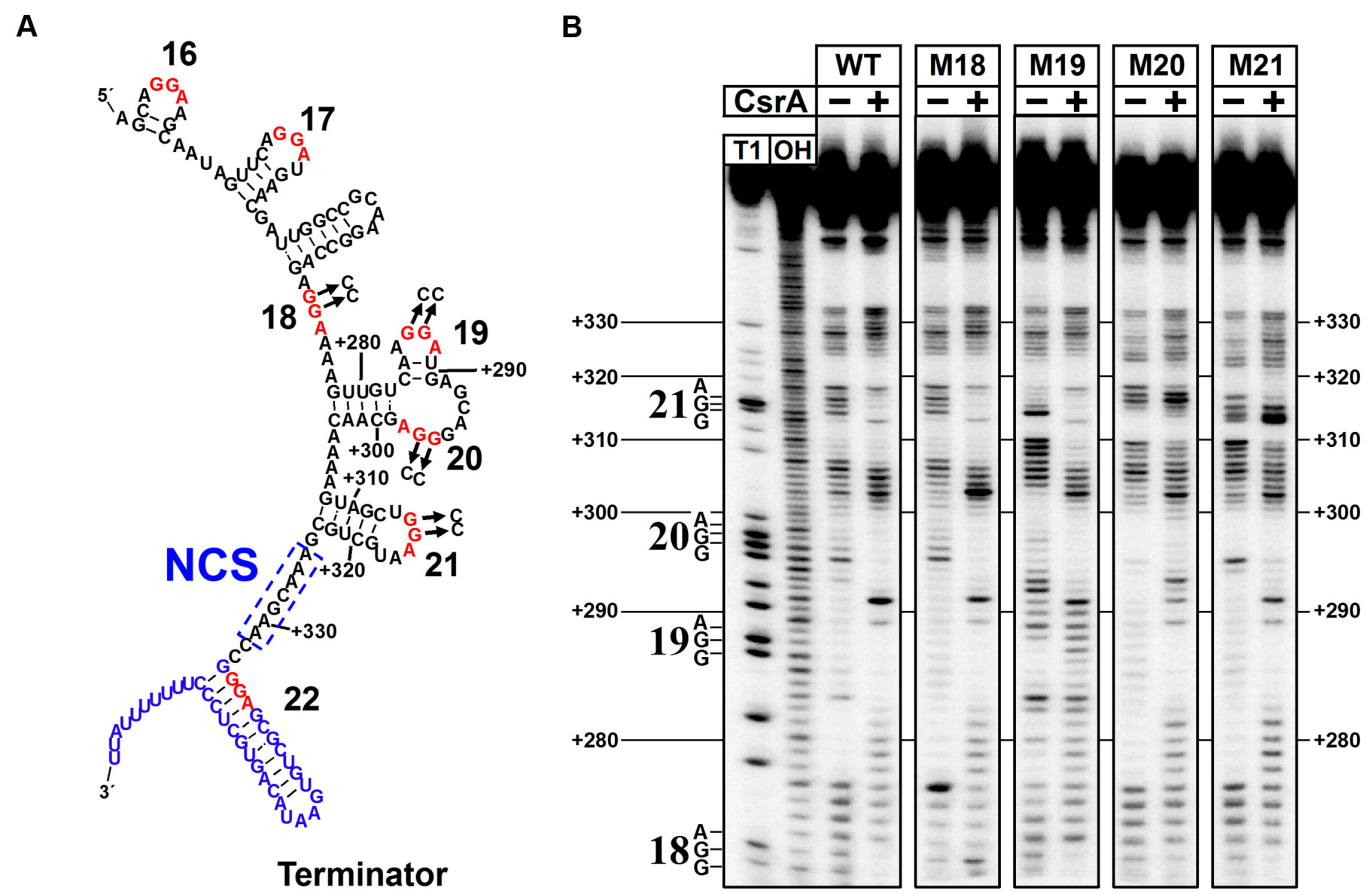
In line probing analysis of CsrA binding to the 3′ terminus of CsrB. (**A**) Structure of the 3′ region of CsrB that encompasses the +226 mutant RNA as determined by in-line probing. The GGA sequences representing potential CsrA binding sites and transcription terminator are indicated with red and blue text, respectively. The NCS is boxed. GGA to CCA mutations were constructed as shown. Numbering is with respect to the full length CsrB sequence. (**B**) In-line probing reactions of the +226 CsrB RNA were performed in the absence and presence of 0.5 μM CsrA. Identical experiments were performed with +226 CsrB WT RNA and mutant RNAs containing GGA to CCA mutations in GGA sites 18, 19, 20 and 21, which are indicated as M18 to M21, respectively. The deduced structure of this region, along with potential CsrA binding sites is indicated to the left. Partial alkaline hydrolysis (OH) and RNase T1 digestion (T1) ladders are shown. Experimental RNAs were analyzed on the same gel but separated digitally, as shown, for clarity.

The in-line probing technique exploits the individual susceptibility of bonds within the RNA backbone to spontaneous cleavage. Low and high cleavage rates reflect rigid versus flexible segments of RNA, respectively, and have been used to infer the structures of riboswitches in the presence and absence of their regulatory ligands (47), Hfq-dependent mRNA–sRNA interactions (53) and protein–RNA interactions of attenuators (54). We therefore sought to use in-line probing to determine the secondary structure of CsrB and to examine CsrA binding to predicted sites near the 3′ end of CsrB. We first analyzed the structure and CsrA binding properties of the full length CsrB RNA using this approach, but found it was difficult to clearly distinguish adjacent bands in the sequencing pattern of this large (369 nt) sRNA (data not shown). To better resolve the CsrB structure and CsrA protection pattern of the region involved in CsrD regulation, we next performed in-line probing experiments with a set of truncated RNAs, including the WT +226 CsrB RNA and four corresponding mutant RNAs that carry GGA to CCA mutations in sites 18, 19, 20 or 21 (Figure 6A). In the absence of CsrA, in-line probing of the WT RNA largely supported the structural prediction of this region. An exception was the predicted loop that carries GGA sites 19 and 20, which was found to be relatively resistant to spontaneous hydrolysis. We therefore suggest a more rigid structure for this region, although other interpretations are possible (compare Figures 5A and 6A).

CsrA strongly protected WT +226 CsrB from spontaneous cleavage at sites 18, 20 and 21, indicating that these are authentic CsrA binding sites (Figure 6B). While site 19 was already relatively resistant to spontaneous cleavage in the absence of CsrA, it became more resistant to cleavage in the presence of CsrA. Furthermore, residues preceding (278–281) and following (289, 291) site 19 became more sensitive to cleavage when CsrA was bound, consistent with them becoming more flexible. Thus, site 19 also appears to represent a CsrA binding site. The site 18 substitution eliminated CsrA protection at site 18 and caused little change in the overall RNA structure in the absence of CsrA. In contrast, the site 19 mutation affected spontaneous cleavage not only at residues overlapping and immediately following site 19, but also at distant downstream locations. For example, residues of the 5′ stem of site 21 (308–310) were destabilized, while residues of the site 21 loop (316–318) were stabilized. The latter result might have been caused by pairing between the mutant site 19 (5′-CCA) with site 21 (5′-UGG), although this was possibility was not formally examined. Similar destabilizing effects in the site 21 stem were also observed for the site 20 and 21 mutations, which also might have resulted from new base pairing interactions between sites 20(mutant):21(WT) and 21(mutant) :20(WT) or 21(mutant):19(WT), respectively. Besides these distinct changes, there are subtle differences in the in-line probing results of the various mutants that may result from long range changes in RNA structure, which we have not attempted to assign.

In all cases but one, GGA to CCA mutations only affected CsrA-dependent protection from spontaneous hydrolysis at that particular site. The lone exception was observed for the mutation of site 20, which eliminated CsrA-dependent protection from cleavage at both sites 20 and 21. Mutation of site 21 did not affect CsrA protection from cleavage at site 20 (Figure 6). These results may suggest that stable binding of a CsrA dimer to GGA site 21 depends on its interaction with GGA site 20 and bridging to the downstream site 21, similar to the CsrA binding mechanism that occurs in the *glgC* and *pnp* mRNA leaders (21,52) and the sequential binding and bridging interactions of the CsrA homolog RsmE that have been mapped in RsmZ sRNA (19).

### CsrA blocks *in vitro* cleavage of CsrB by RNase E

**Figure 7.**
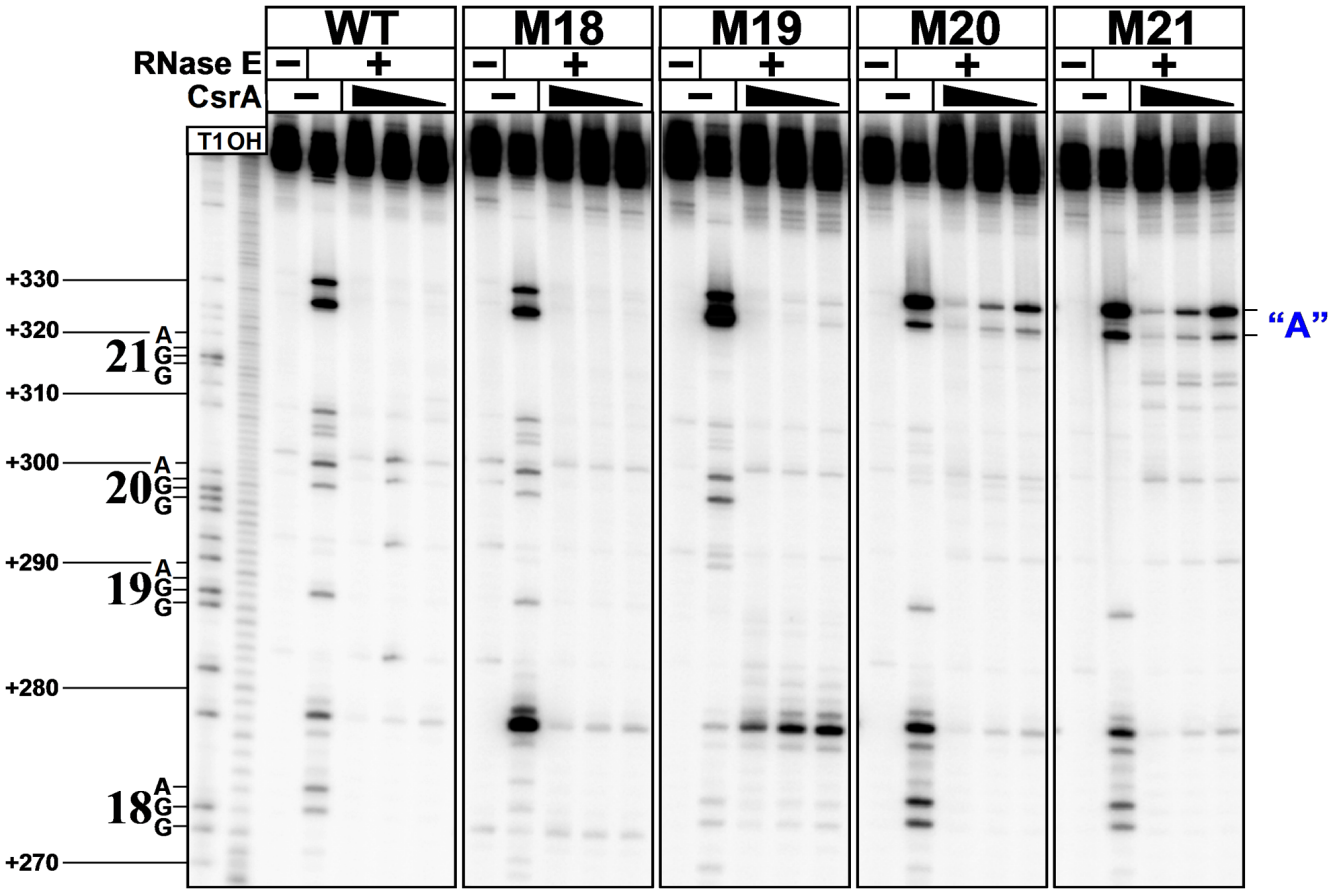
CsrA protects CsrB from *in vitro* RNase E cleavage by binding to GGA sites 20 and 21. *In vitro* cleavage experiments were performed with the +226 CsrB RNA, varying concentrations of CsrA (0.0, 1.0, 0.5 or 0.25 μM) and RNase E (37.5 nM) as indicated. Identical experiments were performed with +226 CsrB RNAs containing GGA to CCA mutations in GGA sites 18, 19, 20 and 21, labeled as M18–M21, respectively. The positions of GGA sites 18–21 and the necessary *in vivo* cleavage location (blue A) that was determined by 3′ RACE (Figure 2) are marked. Control experiments with RNA only are also shown. Partial alkaline hydrolysis (OH) and RNase T1 digestion (T1) ladders are shown. All RNA samples were analyzed on the same gel, but separated digitally for clarity. Numbering is with respect to the full length CsrB sequence.

Taken together with our previously published results (34), our data described above suggest that CsrA protects CsrB from cleavage by RNase E *in vivo* and that the antagonistic effects of CsrA and CsrD on CsrB stability are mediated near the 3′ end of this sRNA. To further probe the mechanism for this regulation, we tested whether RNase E can faithfully recapitulate cleavage of CsrB *in vitro*, and then determined if CsrA can protect CsrB from *in vitro* RNase E attack. We first incubated labeled CsrB RNA with purified RNase E and examined the resulting cleavage pattern. Our results indicated that RNase E cleaves the full length CsrB near nucleotide 280, which is ∼50 bases upstream of the necessary cleavage site mapped *in vivo* (Figure 2 and Supplementary Figure S3). Furthermore, while CsrA protected full length CsrB from cleavage by RNase E *in vitro* (Supplementary Figure S3), mutation of GGA binding sites 20 and 21 did not alter protection by CsrA (data not shown). This finding suggested that the folding pattern of the full length CsrB RNA might differ *in vitro* and *in vivo*. In an attempt to avoid possible aberrant folding and cleavage, we repeated the RNase E cleavage experiments with a shorter fragment of CsrB, +226, whose turnover was nevertheless regulated appropriately by CsrD *in vivo* (Figures 3–5). In this experiment, major RNase E cleavages were seen at nucleotides A327 and A331 (Figure 7). These residues are located in the 3′ segment of CsrB (region ‘A’), the necessary cleavage site (NCS) for *in vivo* decay (Figure 2). The lowest concentration of CsrA tested resulted in near complete protection of +226 CsrB RNA from RNase E-mediated cleavage at both major cleavage sites (Figure 7). Furthermore, mutant RNAs containing small deletions within the NCS (*ΔA*, A1, A2, A3, A4, A5) showed altered *in vitro* cleavage patterns. Most strikingly, *ΔA* and A2 were highly resistant to cleavage, even in the absence of CsrA (Supplementary Figure S4). Mutation of CsrA binding sites 18 and 19 (GGA to CCA), which were largely dispensable for CsrD-dependent decay of CsrB *in vivo* (Figure 4), did not substantially affect CsrA-dependent protection from RNase E cleavage *in vitro* (Figure 7). In contrast, mutation of CsrA binding sites 20 or 21 dramatically reduced the ability of CsrA to protect the +226 CsrB RNA from RNase E cleavage (Figure 7). These results confirmed the *in vivo* evidence that GGA sites 20 and 21 are critically important for CsrA-dependent protection of CsrB, and in the absence of either site, CsrB turnover does not require CsrD. As discussed below, the *in vitro* RNase E cleavage reaction was unaffected by the addition of CsrD in the presence or absence of CsrA.

## DISCUSSION

More than a simple means of disposal, the turnover pathways of sRNAs are often central to their biological roles. Unlike *trans-*acting basepairing sRNAs, which typically require Hfq protein for their function and stability (55), decay of CsrB and CsrC sRNAs of *E. coli* requires the CsrD protein and is unaffected by Hfq (34). The turnover pathways of CsrB/C and many baseparing sRNAs, mRNAs and other RNAs nevertheless share the endonuclease RNase E and the 3′ to 5′ exonuclease PNPase, which degrades RNase E-generated fragments (1,34,56,57). CsrD-mediated decay does not require the C-terminal scaffold domain of RNase E, on which PNPase and other proteins assemble to form the degradosome (34,58,59). Evidence indicates that CsrD is not a nuclease and is not involved in other RNase E-mediated decay pathways that have been examined (34). We recently demonstrated that CsrB/CsrC sRNA decay is activated in response to the presence of glucose by the binding of unphosphorylated EIIA^Glc^ to the EAL domain of CsrD (33). Until now, the reason that RNase E requires CsrD in order to degrade CsrB/C sRNAs was unclear. Our present data show that CsrD is required for CsrB turnover because CsrA binding prevents RNase E from gaining access to the necessary cleavage site (NCS) of this RNA.

Endonucleolytic cleavage of CsrB by RNase E appears to initiate in an unstructured segment located between the last CsrA binding site and the intrinsic terminator, with decay subsequently progressing toward the 5′ end of this RNA (Figures 2, 6, 7). Minimal deletions at the NCS inhibit CsrB turnover (Figure 2). This pattern differs from the generalized transcript decay pattern observed in *E. coli* and from RNase E-mediated turnover of a variety of specific transcripts, which involves endonucleolytic cleavage in the net 5′ to 3′ direction (56,60,61). However, it seems to be consistent with the 3′ to 5′ scanning mechanism observed for RNase E with model RNA substrates (62), and is similar to the decay of certain basepairing RNAs, e.g. GlmZ, in which RNase E initially cleaves just upstream of the terminator (63,64).

RNase E gains access to RNA for turnover by two general mechanisms. The first involves binding to a 5′ monophosphate group and cleavage downstream from it. The alternative ‘direct entry’ route involves RNase E binding close to an internal cleavage site without requiring access to the 5′ end of the transcript (65–68). The former mechanism relies on processing of the original 5′ triphosphate group of newly synthesized RNA to a 5′ monophosphate group by the 5′ pyrophosphohydrolase RppH. CsrB RNA structure likely prevents RppH activity and 5′ entry, which is inhibited by base pairing at the 5′ end of a transcript (69) (Figure 2A). Furthermore, deletion of *rppH* did not have a substantial effect on CsrB decay (Supplementary Figure S5). We presently cannot explain why certain deletions from the 5′ end of CsrB tend to stabilize the resulting truncated transcripts, which nevertheless still require CsrD for efficient turnover (Figure 3).

Recruitment of RNase E can play a key role in the decay pathways of basepairing sRNAs and the mRNAs that they target. Besides exposing mRNA to RNase E passively by inhibiting translation and causing ribosome clearance, an sRNA containing a 5′ monophosphate group can allosterically activate RNase E for mRNA cleavage in a mechanism that appears similar to RNase E activation by the 5′ monophosphate of an mRNA molecule (70,71). In addition, basepairing sRNAs can stabilize their mRNA targets by preventing an initial cleavage or by blocking RNase E progression on a polycistronic transcript (72,73). RNase E cleavage of CsrB and the basepairing sRNA GlmZ share similarity in that they (i) do not involve the 5′ decay route or RppH, (ii) occur in unstructured RNA located immediately upstream from the intrinsic transcription terminator and (iii) require the participation of a non-nucleolytic protein, which renders their decay responsive to cellular physiology. However, the two regulatory mechanisms otherwise differ. The free GlmZ RNA is inaccessible to RNase E cleavage; the binding of RapZ protein to a stem-loop of GlmZ provides a site for recruiting RNase E to gain a foothold on this RNA (64). In contrast, free CsrB RNA is accessible to RNase E cleavage (Figure 7). CsrA binding to CsrB just upstream from the necessary RNase E cleavage site (Figures 2, 6, and 7) may prevent RNase E recruitment to this location before cleaving. Exactly how CsrD permits RNase E access to CsrB remains to be determined. While CsrD binds to CsrB RNA *in vitro*, this binding appears to be non-specific, and CsrD did not facilitate *in vitro* cleavage of CsrA-protected CsrB RNA by RNase E, even in the presence of EIIA^Glc^ (Supplementary Figure S6) (33,34). We suspect that CsrD requires factor(s) in addition to EIIA^Glc^ to render the CsrA-protected CsrB accessible to RNase E cleavage, although this remains to be seen.

The CsrA:CsrB decay inhibitory complex is likely formed by the bridging of a CsrA dimer across the two 3′ terminal binding sites (GGA 20, 21). CsrA possesses two RNA binding surfaces and is known to bridge from a high affinity-binding site to a downstream site that overlaps the Shine-Dalgarno sequences during translational repression (21,52). In addition, structural studies show that the highly conserved CsrA ortholog RsmE binds to its sRNA antagonist RsmZ sequentially from the 5′ to 3′ end, in a cooperative fashion that involves RsmE bridging (19). Importantly, while a GGA to CCA mutation eliminating CsrA binding at the last binding site (GGA 21) in CsrB did not affect binding at the site immediately upstream (GGA 20), a similar mutation of the penultimate site (GGA 20) eliminated binding at GGA 21 (Figure 6), suggestive of ordered bridging from GGA site 20 to 21. The striking loss of the CsrD requirement for turnover of the +284–369 CsrB derivative relative to the slightly longer +263–369 derivative (Figure 3A) was most likely caused by the loss of the double-stranded stem that supports the important GGA 20 site (as well as GGA19). Based on the known binding preference of CsrA for GGA sequences located in hairpin loops (17), such a loss of secondary structure should decrease CsrA binding to GGA 20, causing increased access of RNase E to the NCS. CsrA has also been shown to protect the *flhDC* mRNA against RNase E cleavage by binding to two sites in the 5′ end of this RNA, although CsrA bridging has not been demonstrated in this mechanism (12).

While Csr (Rsm) sRNAs appear to be a defining characteristic of *γ-Proteobacteria* (25,26), CsrD orthologs are found in a limited number of families of this bacterial class, including the *Enterobacteriaceae, Vibrionaceae* and *Shewanellaceae* (34). In *Pseudomonas fluorescens*, a member of the *Pseudomonadaceae* family, which lacks a *csrD* gene, CsrA orthologs protect their sRNA antagonists from degradation (38,39). Because a *csrA* disruption had little or no effect on CsrB/C decay in *E. coli* (23), it seemed that the decay pathway of Csr family sRNAs is quite different in *E. coli* and *P. fluorescens*. In fact, the absence of CsrD caused CsrB turnover in *E. coli* to be inhibited by CsrA, as seen in *P. fluorescens* (Figures 2 and 3).

**Figure 8.**
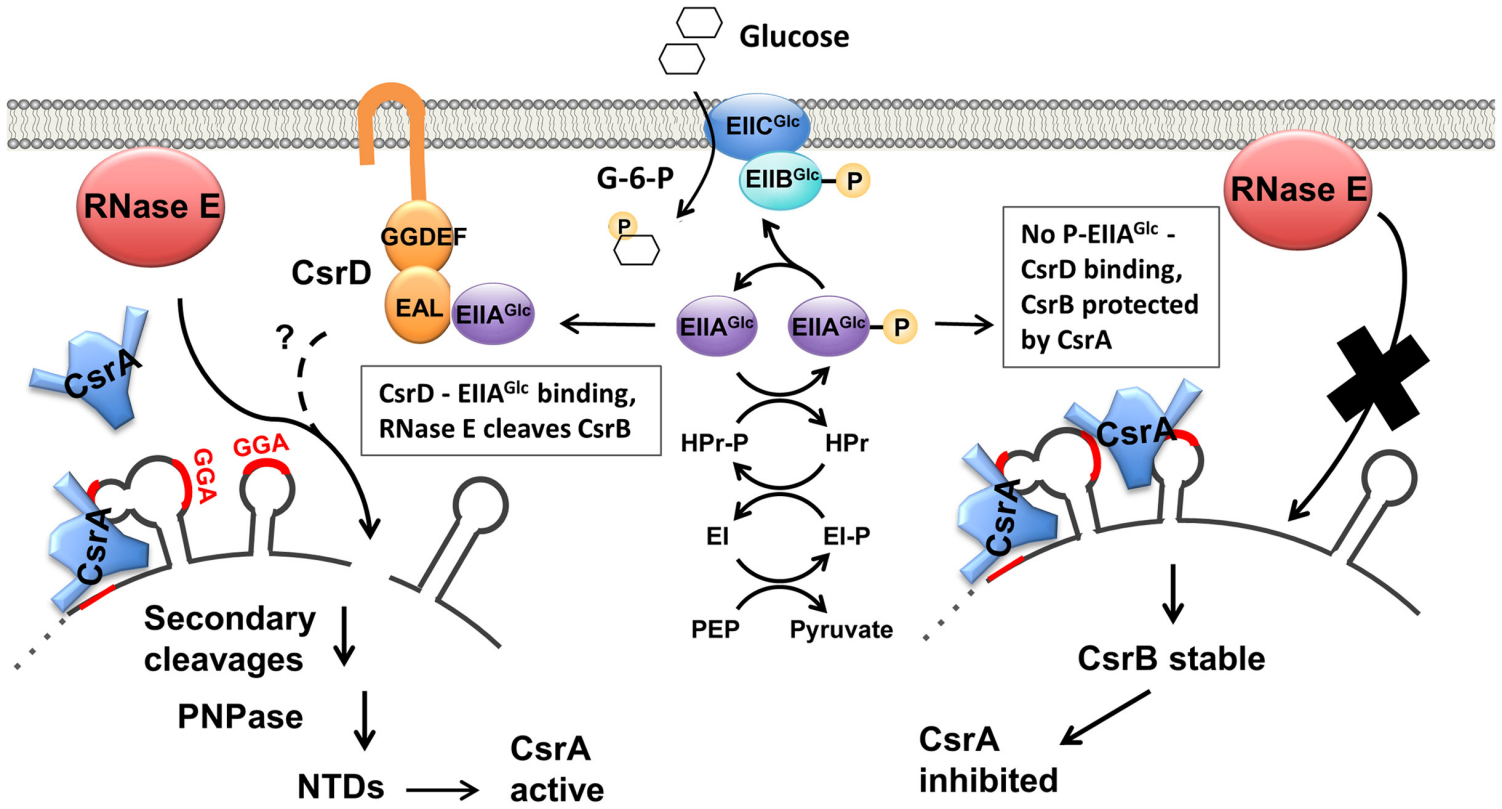
CsrB decay is regulated in response to carbon availability through its effect on CsrA and CsrD antagonism. The phosphorylation state of the PTS protein EIIA^Glc^ serves as an indicator of carbon availability. P-EIIA^Glc^ predominates when a preferred carbon source such as glucose is unavailable, and in this form is unable bind to CsrD. During glucose transport, EIIA^Glc^ becomes dephosphorylated and able to bind to CsrD and potentiate CsrB decay. CsrA binding to CsrB protects it from RNase E cleavage in the absence of CsrD-EIIA^Glc^. A broken line indicates that the molecular mechanism of CsrD remains to be determined. RNase E, PNPase and other nucleases degrade CsrB to nucleotides (NTDs).

By supporting a high rate of CsrB/C decay in the presence of CsrA, and by coupling CsrB/C decay to the availability of preferred carbon source, CsrD serves as an integral part of a regulatory strategy that permits the *E. coli* Csr system to respond rapidly to nutritional cues (31,33,74,75). CsrD helps to insure that CsrB/C levels are kept low when carbon resources are optimal and CsrA is needed to activate the expression of genes and pathways needed for rapid growth, e.g. glycolysis (Figure 8). Conversely, when preferred carbon substrate has been expended and metabolic end products such as formate and acetate are present, the BarA-UvrY TCS will activate CsrB/C synthesis and CsrD-dependent turnover will decrease. The resulting accumulation of CsrB/C will cause CsrA sequestration and the induction of stationary phase and stress resistance pathways that are negatively regulated by CsrA. Because in *P. fluorescens* the Csr-family sRNAs decay slowly (38,39) and are not regulated by CsrD, changes in their levels should be less rapidly responsive to nutritional or other environmental changes, although this idea has not been explicitly tested. Our studies suggest that CsrD evolved from a prototypical GGDEF-EAL protein to serve as a means of decoupling Csr sRNA decay from CsrA binding and receiving information about carbon nutritional status from the PTS pathway for transmission to the Csr sRNA decay pathway. We propose that this evolutionary step improved both the regulatory flexibility and response time of the Csr system, which depend upon CsrD and a negative feedback loop of the Csr system (27,75). How this shift in the decay process for Csr (Rsm) sRNA turnover in a subset of γ-proteobacterial families relates to the diverse metabolic and regulatory strategies employed by species in this important class of bacteria remains to be determined.

## Supplementary Material

SUPPLEMENTARY DATA
