# Supplementary material for: Antagonistic control of the turnover pathway for the global regulatory sRNA CsrB by the CsrA and CsrD proteins
Source: Nucleic Acids Res. 2016 May 27;44(16):7896–910. doi: 10.1093/nar/gkw484 (PMC5027483; doi:10.1093/nar/gkw484)
Supplement: SUPPLEMENTARY DATA [file supp_44_16_7896__index.html]

Antagonistic control of the turnover pathway for the global regulatory sRNA CsrB by the CsrA and CsrD proteins — Antagonistic control of the turnover pathway for the global regulatory sRNA CsrB by the CsrA and CsrD proteins — SUPPLEMENTARY DATA 

# Antagonistic control of the turnover pathway for the global regulatory sRNA CsrB by the CsrA and CsrD proteins

## SUPPLEMENTARY DATA

- SUPPLEMENTARY DATA
