# Supplementary material for: Antagonistic control of the turnover pathway for the global regulatory sRNA CsrB by the CsrA and CsrD proteins
Source: Nucleic Acids Res. 2016 May 27;44(16):7896–910. doi: 10.1093/nar/gkw484 (PMC5027483; doi:10.1093/nar/gkw484)
Supplement: SUPPLEMENTARY DATA [file supp_gkw484_nar-00473-y-2016-File010.pdf]

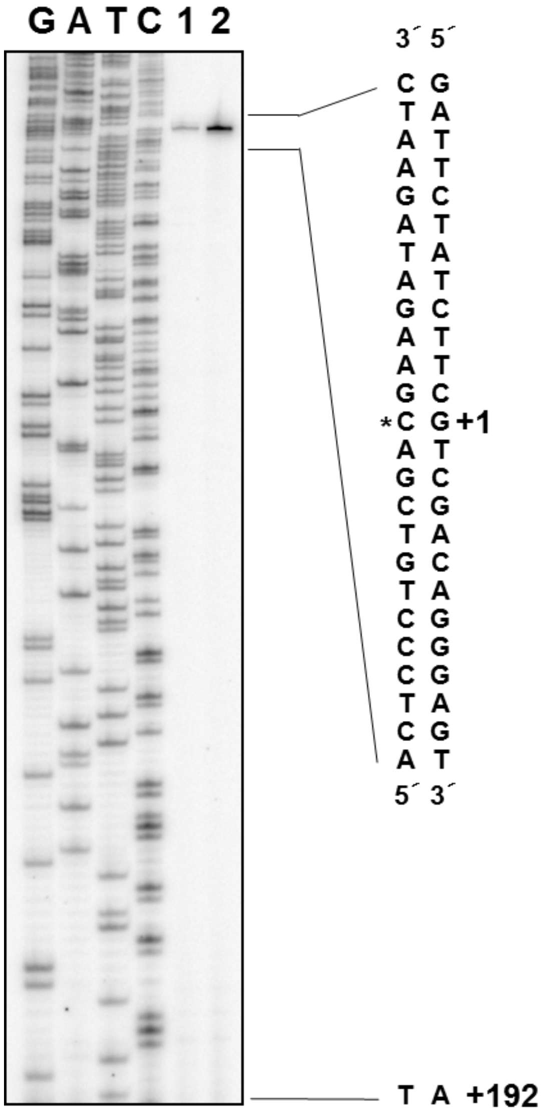

Supplementary Figure S1. Primer extension analysis of CsrB from wild type and *pnpΔ683* mutant strains. Steady-state RNA was analyzed from bacterial cultures grown to the transition from the exponential to the stationary phase of growth. Products from *pnpΔ683* and wild-type transcripts are shown in lanes 1 and 2, respectively. The dideoxy-sequencing ladder (lanes G, A, T and C) was generated with PEX7 using pCSRB-SF as a template.

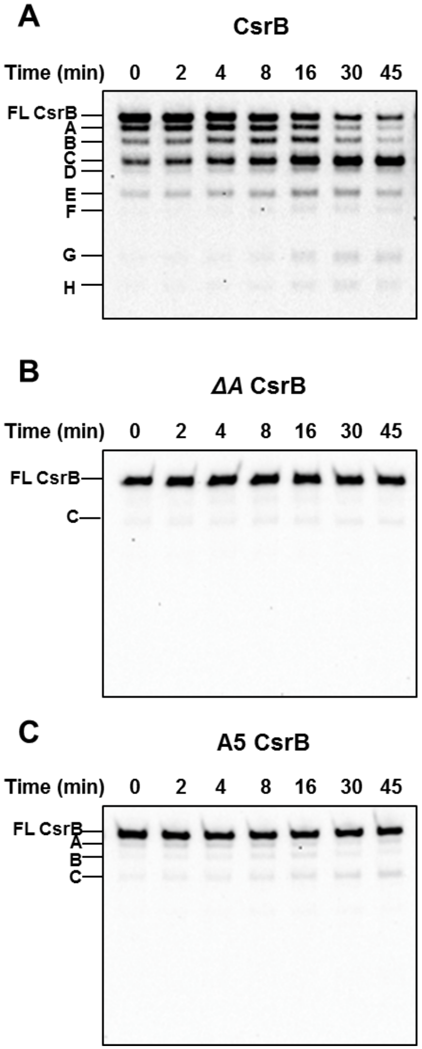

Supplementary Figure S2. Mutations in the NCS region stabilize CsrB in the absence of PNPase. *E. coli* strain SK10019 (*Δpnp683*) was deleted for *csrB* at its native chromosomal locus and expresses the wild-type CsrB RNA (A), *ΔA* CsrB (B), and A5 CsrB (C) under *araP*$_{BAD}$ transcriptional control from the phage λ attachment site. Expression was induced by addition of arabinose at the transition to stationary phase of growth (OD_{600} of 1.0), and rifampicin RNA decay assays were performed 20 min following induction. The 23S and 16S rRNAs were detected by methylene blue staining and served as RNA loading controls (not shown). Half-lives were calculated by non-linear regression analysis of best-fit decay curves. The half-life of full length (FL) RNA in strains expressing WT, *ΔA*, and A5 CsrB RNAs was 14 min, >45 min, and >45 min, respectively. See Figure 2C of the main text for description of mutant CsrB RNAs.

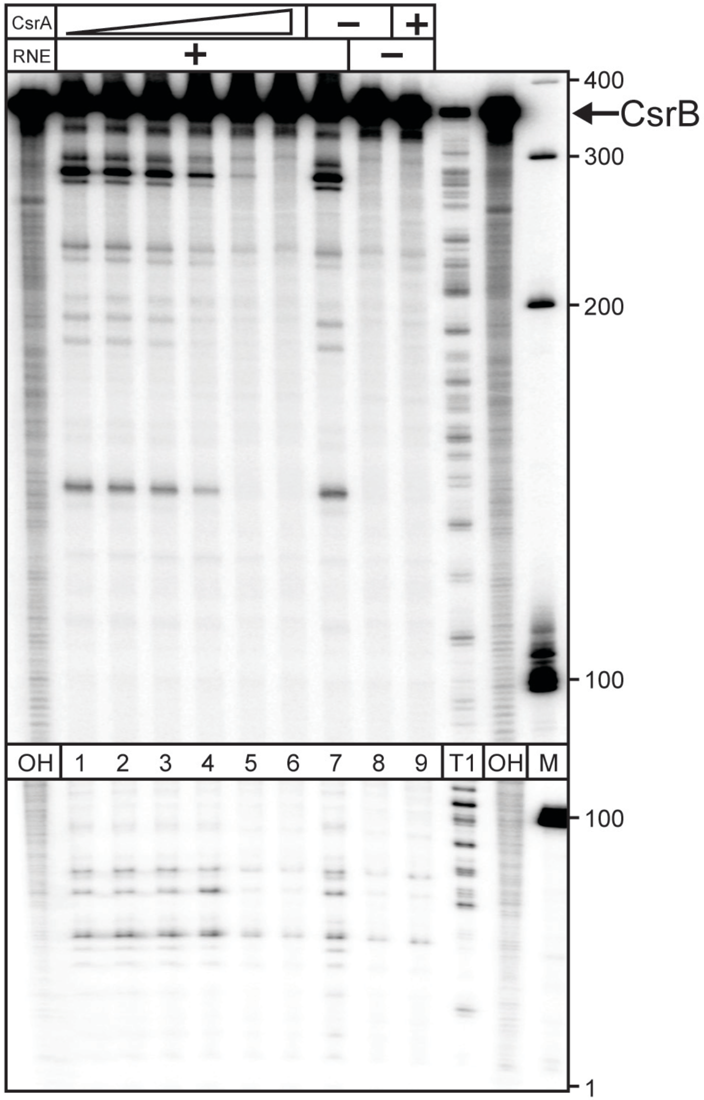

Supplementary Figure S3. CsrA protects the full-length CsrB RNA from cleavage by RNase E. Cleavage experiments were performed with CsrB RNA, several concentrations of CsrA protein (0.0625, 0.125, 0.25, 0.5, 1.0, and 2.0 μM), and RNase E (37.5 nM) and analyzed by denaturing electrophoresis. Sequence identification ladders were generated with RNase T1 (T1) and alkaline hydrolysis (OH). RNA standards (M) were separated alongside CsrB to mark the 300, 200, 100, and 10 nt positions. The reactions were run on a single sequencing gel with multiple loadings. The 5’ segment of CsrB (top image) originates form the initial loading while the 3’ segment (bottom image) originates from the final loading.

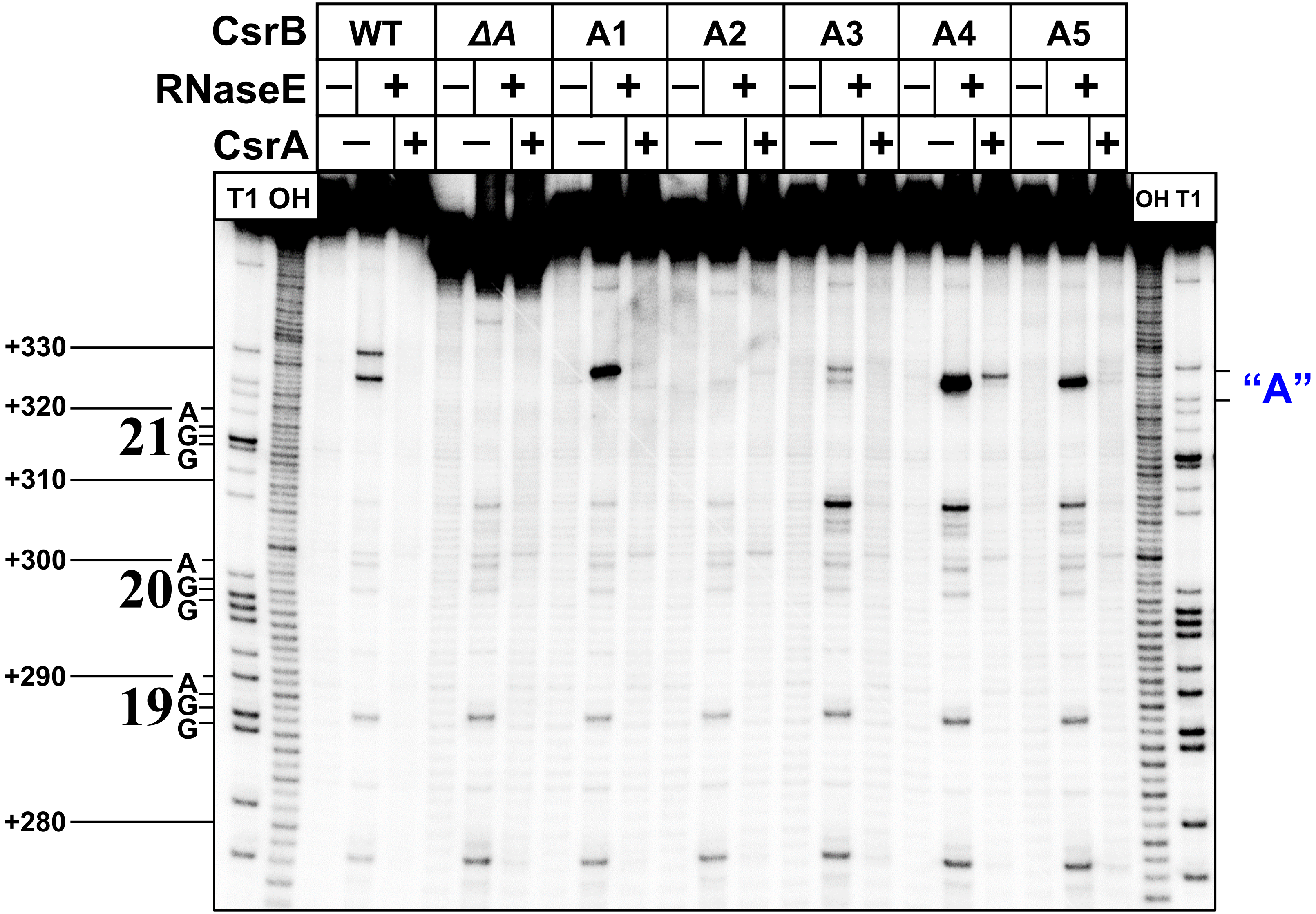

Supplementary Figure S4. Mutations in the NCS affect RNase E-dependent decay of CsrB *in vitro*. *In vitro* cleavage experiments were performed with the +226 CsrB RNA, 0.5 μM CsrA, and 37.5 nM RNase E. Identical experiments were performed with +226 CsrB RNAs containing NCS mutations *ΔA*, A1, A2, A3, A4, and A5. The necessary *in vivo* cleavage location ("A") that was determined by 3' RACE (Figure 2) is marked. Control experiments with RNA only and partial alkaline hydrolysis (OH) and RNase T1 digestion (T1) ladders are shown. Numbering is with respect to the full length CsrB sequence.

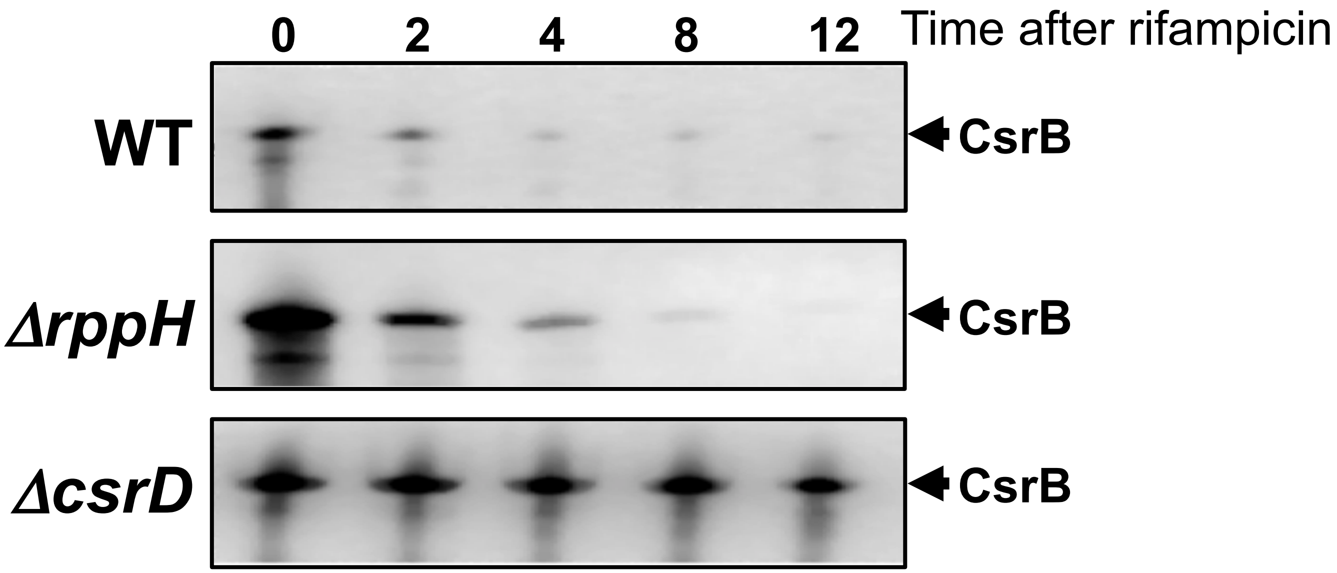

Supplementary Figure S5. Deletion of *rppH* does not affect CsrB decay. Wild-type (WT), *ΔrppH*, or *ΔcsrD* deletion strains were grown to the mid-exponential phase of growth in LB at 37° C, and total RNA was isolated at the indicated time points past rifampicin addition. Northern blots were performed using anti-sense CsrB riboprobes.

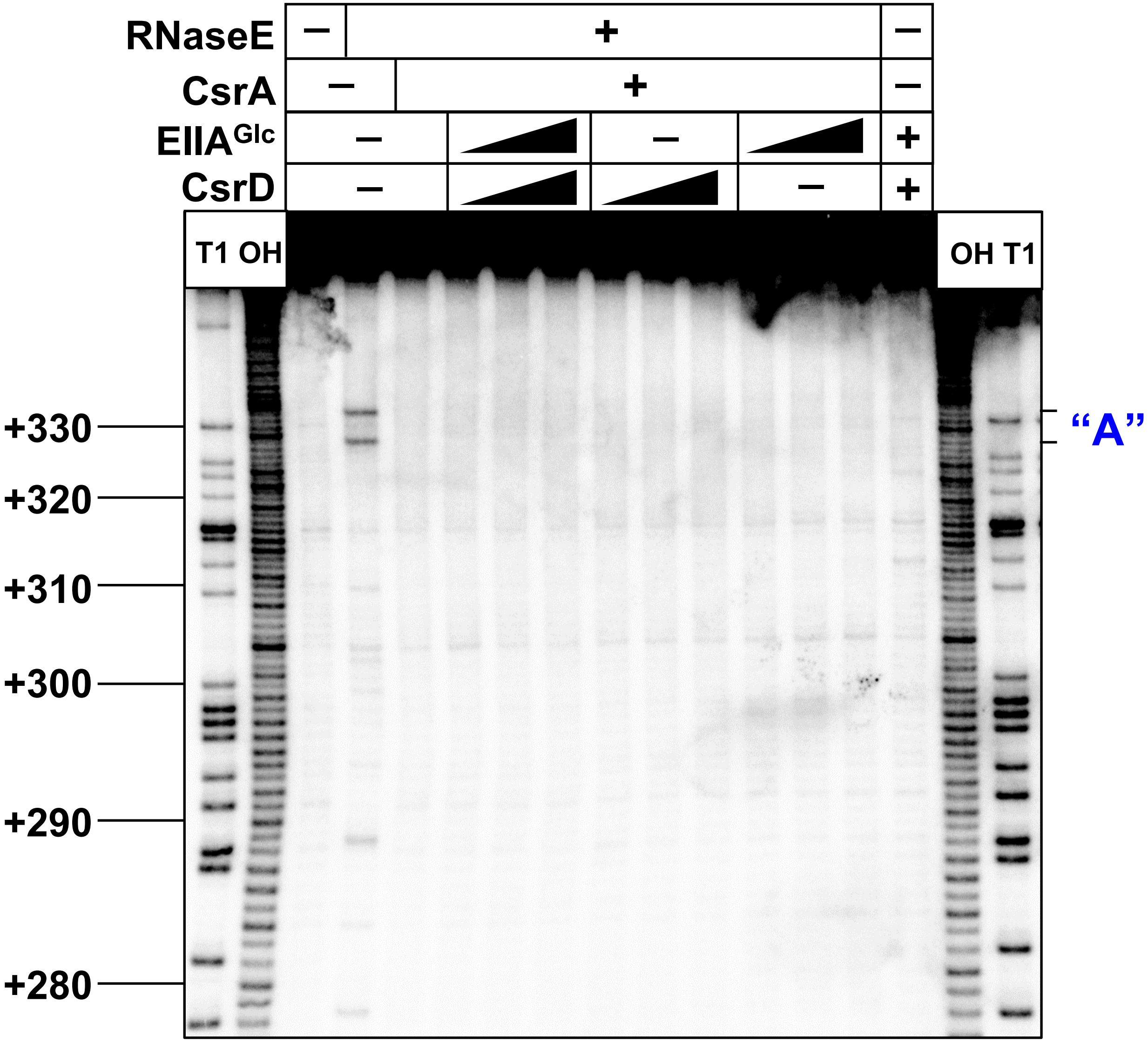

Supplementary Figure S6. Effect of $EIIA^{Glc}$ and CsrD on RNase E-dependent cleavage of CsrB *in vitro*. These experiments were performed with the +226 CsrB RNA, 0.5 μM CsrA, 37.5 nM RNase E, $EIIA^{Glc}$ (0.5, 2 or 6 μM), and CsrD tetramer (0.125, 0.5 or 1.5 μM) as indicated. Premixed $EIIA^{Glc}$-CsrD complex or each protein individually was added to reactions and incubated at 25 °C for 10 min. CsrA and RNase E were then sequentially added to reactions and cleavage was allowed to proceed for 10 min at 25 °C. Reactions were stopped and analyzed as described in Materials and Methods. Control experiments were performed with RNA only. Partial alkaline hydrolysis (OH) and RNase T1 digestion (T1) ladders are also indicated. Numbering is with respect to the full length CsrB sequence.

Table S1 – Bacterial Strains

Strain	Description	Reference
MG1655	Prototrophic E. coli K12	Michael Cashel
MG1655 *ΔcsrB*	MG1655 with marked *csrB* deletion - Cam^r	(1)
BL21 attλ::DE3 TUNER	Protein purification strain carrying the DE3 λ lysogen	Agilent Technologies
MG1655 *ΔcsrD*	MG1655 with unmarked *csrD* deletion	(2)
MG1655 *csrA::kan*	MG1655 with *csrA* disrupted after amino acid position 50 – KnR	(3)
MG1655 *csrA::gm*	MG1655 with *csrA* disrupted after amino acid position 50 - GmR	This study
MG1655 *ΔrppH*	MG1655 with unmarked *rppH* deletion (moved with transduction)	(4)
RGKSB837	Δ*lacZ*(MluI) Δ(*att-lom*)::*bla* Φ(*csrB-lacZ*)1(Hyb) Δ*csrB*::*cam*	(1)
MG1693	*thyA715 rph-1*	*E. coli* Genetic Stock Center
SK10019	*pnpΔ683*::str^r/spc^r *thyA715 rph-1*	(5)
SK10019 *ΔcsrB*	SK10019 with an umarked *csrB* deletion	This study

Table S2 – Plasmids

Plasmid Name	Description	Relevant Primers	Reference
pBR322	ectopic expression of proteins - Amp^r Tet^r	N/A	(6)
pKD46	Arabinose-inducible Red recombinase expression vector - Amp^r	N/A	(7)
pAH125	CRIM plasmid containing phage λ integration attachment sites - Kan^r	N/A	(8)
pET24a	CTD-6xHIS protein expression and purification plasmid - Kan^r	N/A	this study
pCsrB	CsrB Expression clone used in Figure 2	(EcoRIF-EcoRIR)	this study
pCsrB-*ΔA*	pCsrB with a deletion from A325 to C333 in *csrB* made by site directed mutagenesis	(3TER-F-3TER-R)	this study
pCsrB-A1	pCsrB with a deletion of A325 and A326 in *csrB* made by site directed mutagenesis	(Δ325-326F-Δ325-326R)	this study
pCsrB-A2	pCsrB with a deletion of C328 to A331 in *csrB* made by site directed mutagenesis	(Δ328-331F-Δ328-331R)	this study
pCsrB-A3	pCsrB with a deletion of C328 to G329 in *csrB* made by site directed mutagenesis	(Δ328-329F-Δ328-329R)	this study
pCsrB-A4	pCsrB with a deletion of G329 to A330 in *csrB* made by site directed mutagenesis	(Δ329-330F-Δ329-330R)	this study
pCsrB-A5	pCsrB with a deletion of A330 to A331 in *csrB* made by site directed mutagenesis	(Δ330-331F-Δ330-331R)	this study
P1vr22	pET24a derivative with the *rne* gene cloned in frame with CTD 6xHIS - Kan^r	(Rnehis5nde-rnechis3ecor)	this study
p1VR147f	pAH125 derivative used for integration of P_{araB}-CsrB in *att*λ – Kan^r	(usparaCPBKpn-dsparaCBlunt)-(uscsrBblunt-dscsrBxbaI)	this study
p1VR147c	pAH125 derivative used for integration of P_{araB}-CsrB*Δ1-150* in *att*λ - Kan^r	(usparaCPBKpn-dsparaCBlunt)-(csrBtrunc150-dscsrBxbaI)	this study
p1VR147d	pAH125 derivative used for integration of P_{araB}-CsrB*Δ1-225* in *att*λ - Kan^r	(usparaCPBKpn-dsparaCBlunt)-(csrBtrunc225-dscsrBxbaI)	this study
p1VR147g	pAH125 derivative used for integration of P_{araB}-CsrB*Δ1-243* in *att*λ – Kan^r	(usparaCPBKpn-dsparaCBlunt)-(csrBtrunc243-dscsrBxbaI)	this study
p1VR147h	pAH125 derivative used for integration of P_{araB}-CsrB*Δ1-262* in *att*λ - Kan^r	(usparaCPBKpn-dsparaCBlunt)-(csrBtrunc262-dscsrBxbaI)	this study
p1VR147i	pAH125 derivative used for integration of P_{araB}-CsrB*Δ1-283* in *att*λ - Kan^r	(usparaCPBKpn-dsparaCBlunt)-(csrBtrunc283-dscsrBxbaI)	this study
p2VR112	*csrA* gene cloned into the EcoRI-BamHI sites of pBR322 - ApR	(uscsrA300ecoRI-dscsrAbamHI)	this study
p1VR147f-GGA16	p1VR147f with GGA site "16" changed to CCA by site directed mutagenesis	(csrBGGCC1top-csrBGGCC1btm)	this study
p1VR147f-GGA17	p1VR147f with GGA site "17" changed to CCA by site directed mutagenesis	(csrBGGCC2top-csrBGGCC2btm)	this study
p1VR147f-GGA18	p1VR147f with GGA site "18" changed to CCA by site directed mutagenesis	(csrBGGCC3top-csrBGGCC3btm)	this study
p1VR147f-GGA19	p1VR147f with GGA site "19" changed to CCA by site directed mutagenesis	(csrBGGCC4top-csrBGGCC4btm)	this study
p1VR147f-GGA20	p1VR147f with GGA site "20" changed to CCA by site directed mutagenesis	(csrBGGCC5top-csrBGGCC5btm)	this study
p1VR147f-GGA21	p1VR147f with GGA site "21" changed to CCA by site directed mutagenesis	(csrBGGCC6top-csrBGGCC6btm)	this study
p1VR147f-GGA22	p1VR147f with GGA site "22" changed to CCA by site directed mutagenesis	(csrBGGCC7partAtop-csrBGGCC7partAbtm)	this study
p1VR147f-GGA22term	p1VR147f-GGA22 with terminator stem CC changed to GG by site directed mutagenesis	(csrBGGCC7partBtop-csrBGGCC7partBbtm)	this study
pCSRB0AF	*csrB* in pUC19 used in Supplementary Figure 1	N/A	(9)
p1VR147f-*ΔA*	p1VR147f with a deletion from A325 to C333 in *csrB*	(phospAHCsrB ΔA/A5F-pAHCsrBΔA-R)	This study
p1VR147f-A5	p1VR147f with a deletion of A330 to A331 in *csrB*	(hospAHCsrB ΔA/A5F-pAHCsrBA5-R)	This study

Table S3 – Primers (Plasmid Construction)

Name	(Restriction Site) Sequence
rnehis5nde	CAAGTACCGCATATGAAAAGAATGTTAATCAACGCAACTCAGCAG
rnechis3ecor	CAAGTACCGGAATTCGGCTCAACAGGTTGCGGACGCGCAGGAGCGGC
EcoRIF	GCCGCCGAATTCTAATCCAAATACCCCATCTGGTTG
EcoRIR	GCCGCCGAATTCAAACTGCCGCGAAGGATAGCAG
3TER-R	TTCACAGCGCTCCCCGCAGCATTCCAG
3TER-F	CTGGAATGCTGCGGGGAGCGCTGTGAA
Δ325-326F	GTAGCTGGAATGCTGCGACGAACCGGGAGCGCTG
Δ325-326R	CAGCGCTCCCGGTTCGTCGCAGCATTCCAGCTAC
Δ328-331F	GTAGCTGGAATGCTGCGAAACCGGGAGCGCTGTGAATAC
Δ328-331R	GTATTCACAGCGCTCCCGGTTTCGCAGCATTCCAGCTAC
Δ328-329F	GTAGCTGGAATGCTGCGAAAAACCGGGAGCGCTGTG
Δ328-329R	CACAGCGCTCCCGGTTTTTCGCAGCATTCCAGCTAC
Δ329-330F	GCTGGAATGCTGCGAAACACCGGGAGCGCTGTGAATAC
Δ329-330R	GTATTCACAGCGCTCCCGGTGTTTCGCAGCATTCCAGC
Δ330-331F	CTGGAATGCTGCGAAACGCCGGGAGCGCTGTGAATAC
Δ330-331R	GTATTCACAGCGCTCCCGGCGTTTCGCAGCATTCCAG
usparaCPBKpn	ACCGACCAGGGTACCCATCGATTTATTATGACAACTTGAC
dsparaCBlunt	/5Phos/ATGGAGAAACAGTAGAGAGTTGCGA
uscsrBblunt	GTCGACAGGGAGTCAGACAACGAAG
dscsrBxbaI	ACAGAGCAGTCTAGAAAAAACTGCCGCGAAGGATAGCAGG
csrBtrunc150	AACCGGTCAGGATGATTCGGTGGGT
csrBtrunc225	AGCAGGAAGCAATAGTTCAGGATGA
csrBtrunc243	AGGATGAACGATTGGCCGCAAGGCC
csrBtrunc262	AAGGCCAGAGGAAAAGTTGTCAAGG
csrBtrunc283	AAGGATGAGCAGGGAGCAACAAAAG
uscsrA300ecoRI	ACAGTAACAGAATTCGATGCTGCGGCCTTACCTGCAGCGT
dscsrAbamHI	ACAGTAACAGGATCCTTAGTAACTGGACTGCTGGGATTTT
csrBGGCC1top	GGGGTGGTGTGAGCACCAAGCAATAGTTCA
csrBGGCC1btm	TGAACTATTGCTTGGTGCTCACACCACCCC
csrBGGCC2top	AAGCAATAGTTCACCATGAACGATTGGCCG
csrBGGCC2btm	CGGCCAATCGTTCATGGTGAACTATTGCTT
csrBGGCC3top	GGCCGCAAGGCCAGACCAAAAGTTGTCAA
csrBGGCC3btm	TTGACAACTTTTGGTCTGGCCTTGCGGCC
csrBGGCC4top	AAAAGTTGTCAACCATGAGCAGGGAGCAAC
csrBGGCC4btm	GTTGCTCCCTGCTCATGGTTGACAACTTTT
csrBGGCC5top	GTCAAGGATGAGCAGCCAGCAACAAAAGTAGC
csrBGGCC5btm	GCTACTTTTGTTGCTGGCTGCTCATCCTTGAC
csrBGGCC6top	GCAACAAAAGTAGCTCCAATGCTGCGAAACGA
csrBGGCC6btm	TCGTTTCGCAGCATTGGAGCTACTTTTGTTGC
csrBGGCC7partAtop	CTGCGAAACGAACCGCCAGCGCTGTGAATACA
csrBGGCC7partAbtm	TGTATTCACAGCGCTGGCGGTTCGTTTCGCAG
csrBGGCC7partBtop	TGTGAATACAGTGCTGGCTTTTTTTATT
csrBGGCC7partBbtm	AATAAAAAAAGCCAGCACTGTATTCACA
phospAHCsrB ΔA/A5F	/phos/GGGAGCGCTGTGAATACAGTGCTCC
pAHCsrBΔA-R	CGCAGCATTCCAGCTACTTTTGTTG
pAHCsrBA5-R	GGCGTTTCGCAGCATTCCAGCTACT

Table S3 – Miscellaneous

Name	Sequence	Function
T7FLcsrBstart3G	TAATACGACTCACTATAGGGTCGACAGGGAGTCAGACAACGAAG	T7 *in vitro* transcription CsrB template (full length)
T7226csrBstart3G	TAATACGACTCACTATAGGGAGCAGGAAGCAATAGTTCAGGATG	T7 *in vitro* transcription CsrB template (+226-369)
csrBdsT7ter	AATAAAAAAAGGGAGCACTGTATT	3’ CsrB *in vitro* transcription template
ASsrB3	GTAATACGACTCACTATAGGGATTCACAGCGCTCCCGGTTCGTTTCGCA	Anti-sense CsrB Northern Probe (REV-FL)
csrBR	GCGTTAAAGGACACCTCCAGG	Anti-sense CsrB Northern Probe (FWD-FL)
T7CsrB5	GTAATACGACTCACTATAGGCCAGAAGCCTTTCCCTGAAACAC	Anti-sense 5’-CsrB Northern Probe (REV-5’)
CsrB5F	GAGTCAGACAACGAAGTGAACATC	Anti-sense 5’-CsrB Northern Probe (FWD-5’)
T7CsrB3	GTAATACGACTCACTATAGGCAGCGCTCCCGGTTCGTTTCGCAG	Anti-sense 3’-CsrB Northern Probe (REV-3’)
CsrB3F	GCAAGGCCAGAGGAAAAGTTGTC	Anti-sense 3’-CsrB Northern Probe (FWD-3’)
IdT3’RACE	AGCATGCGGCCGCTAAGAACAGTG	3’ RACE CsrB amplification primer (FWD)
CsrB3’RACE	GAGTCAGACAACGAAGTGAACATCAG	3’ RACE CsrB amplification primer (REV)
csrAgmUS	TACTGATGAATCCCCTAATGATTTTGGTAAAAATCATTAATTGACAATTAATCATCGGCT	construction of *csrA::gm*
gentUS	TTGACAATTAATCATCGGCTCGTATAATGTGTGGAATTAGCTTAGGATCTAGAATTCTATGTTACGCAGCAGCAACGA	construction of *csrA::gm*
gentcsrADS	TGAGGGTGCGTCTCACCGATAAAGATGAGACGCGGAAAGATTAGGTGGCGGTACTTGGGT	construction of *csrA::gm*
PEX7	ACCACCCCGATGTGATACTTC	Primer extension

References

1. Gudapaty, S., Suzuki, K., Wang, X., Babitzke, P. and Romeo, T. (2001) Regulatory interactions of Csr components: the RNA binding protein CsrA activates *csrB* transcription in *Escherichia coli*. *J. Bacteriol.*, **183**, 6017-6027.
2. Suzuki, K., Babitzke, P., Kushner, S.R. and Romeo, T. (2006) Identification of a novel regulatory protein (CsrD) that targets the global regulatory RNAs CsrB and CsrC for degradation by RNase E. *Genes Dev.*, **20**, 2605-2617.
3. Romeo, T., Gong, M., Liu, M.Y. and Brun-Zinkernagel, A.M. (1993) Identification and molecular characterization of *csrA*, a pleiotropic gene from *Escherichia coli* that affects glycogen biosynthesis, gluconeogenesis, cell size, and surface properties. *J. Bacteriol.*, **175**, 4744-4755.
4. Baba, T., Ara, T., Hasegawa, M., Takai, Y., Okumura, Y., Baba, M., Datsenko, K.A., Tomita, M., Wanner, B.L. and Mori, H. (2006) Construction of *Escherichia coli* K-12 in-frame, single-gene knockout mutants: the Keio collection. *Mol. Syst. Biol.,* **2**, 2006-0008.
5. Mohanty, B.K. and Kushner, S.R. (2003) Genomic analysis in *Escherichia coli* demonstrates differential roles for polynucleotide phosphorylase and RNase II in mRNA abundance and decay. *Mol. Microbiol.*, **50**, 645-658.
6. Bolivar, F., Rodriguez, R.L., Greene, P.J., Betlach, M.C., Heyneker, H.L., Boyer, H.W., Crosa, J.H. and Falkow, S. (1997) Construction and characterization of new cloning vehicles. II. A multipurpose cloning system. *Gene,* **2**, 95–113.
7. Datsenko, K.A. and Wanner, B.L. (2000) One-step inactivation of chromosomal genes in *Escherichia coli* K-12 using PCR products. *Proc. Natl. Acad. Sci. U. S. A.*, **97**, 6640-6645.
8. Haldimann, A. and Wanner, B.L. (2001) Conditional-replication, integration, excision, and retrieval plasmid-host systems for gene structure-function studies of bacteria. *J. Bacteriol.*, **183**, 6384-6393.
9. Liu, M.Y., Gui, G., Wei, B., Preston, J.F., 3rd, Oakford, L., Yuksel, U., Giedroc, D.P. and Romeo, T. (1997) The RNA molecule CsrB binds to the global regulatory protein CsrA and antagonizes its activity in *Escherichia coli*. *J. Biol. Chem.*, **272**, 17502-17510.
